# Supplementary material for: Inhibition of WAVE Regulatory Complex Activation by a Bacterial Virulence Effector Counteracts Pathogen Phagocytosis
Source: Cell Rep. 2016 Oct 11;17(3):697–707. doi: 10.1016/j.celrep.2016.09.039 (PMC5081413; doi:10.1016/j.celrep.2016.09.039)
Supplement: Document S1. Figures S1–S5 [file mmc1.pdf]

Cell Reports, Volume *17*

Supplemental Information

Inhibition of WAVE Regulatory Complex Activation by a Bacterial Virulence Effector Counteracts Pathogen Phagocytosis

Daniel Humphreys, Vikash Singh, and Vassilis Koronakis

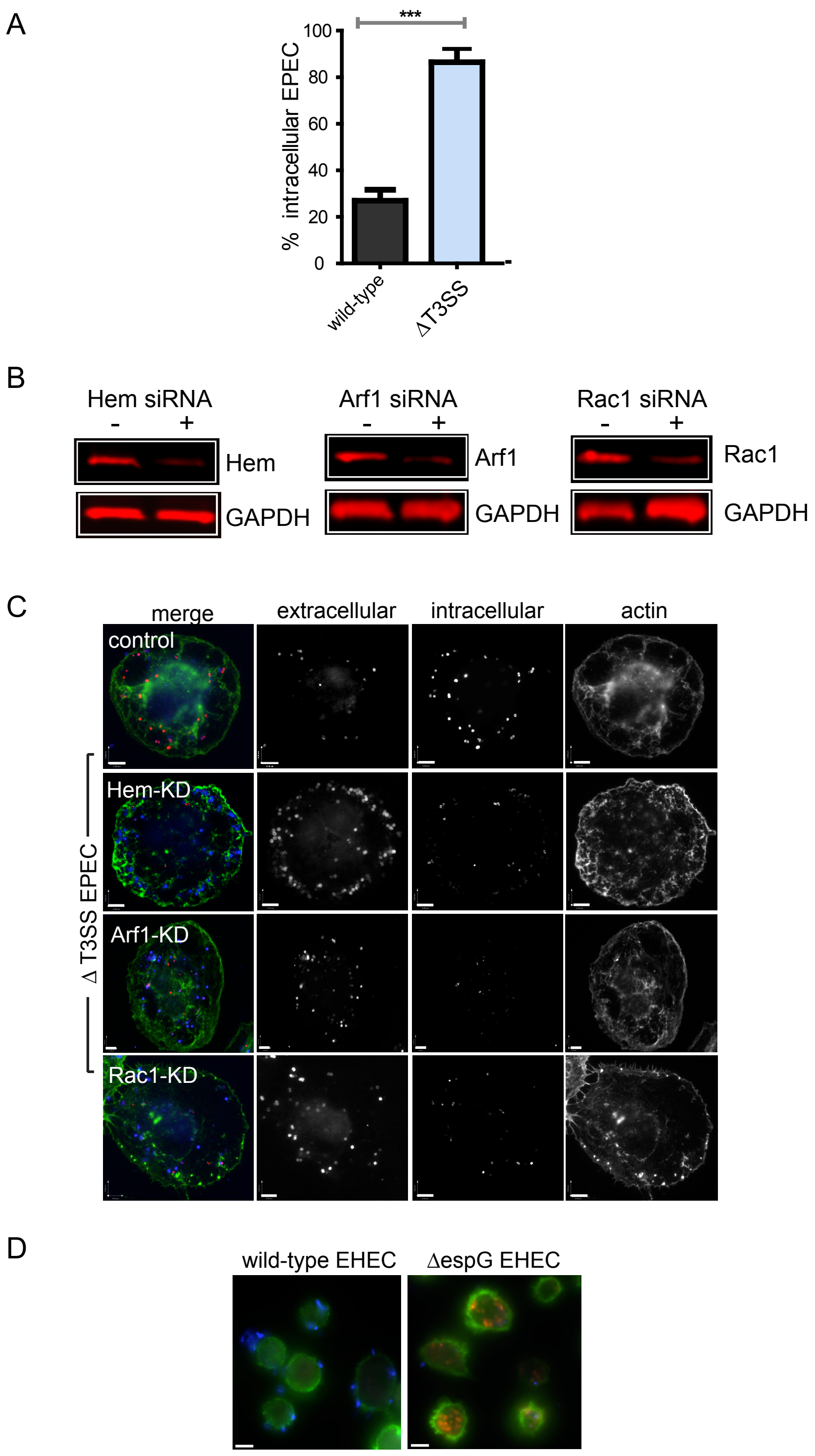

Figure S1. Anti-phagocytosis of EPEC and EHEC (related to Fig 1).

(**A**) Phagocytosis of wild-type and ΔT3SS EPEC in RAW264.7 mouse macrophage cells. Error bars represent ± SEM. Asterisks indicate a significant difference from wild-type. (**B**) Knockdown of Hem, Arf1 and Rac1 in THP1s following siRNA transfection from Figure 1. Immunoblotting of whole cell lysates with antibodies recognising Hem, Arf1 and Rac1 and GAPDH as a loading control. (**C**) Images showing phagocytosis of $EPEC^{\Delta T3SS}$ (ΔT3SS) in knockdown cells from (B). Intracellular bacteria (red), extracellular bacteria (blue) and host cells (actin). Scale bars 6μm. Quantification shown in Fig 1C. (**D**) Phagocytosis of wild-type or ΔespG EHEC by THP1s. Intracellular bacteria (red), extracellular bacteria (blue) and host cells (actin). Scale bars 6μm. All experiments in Fig S1 were performed in triplicate at least three times.

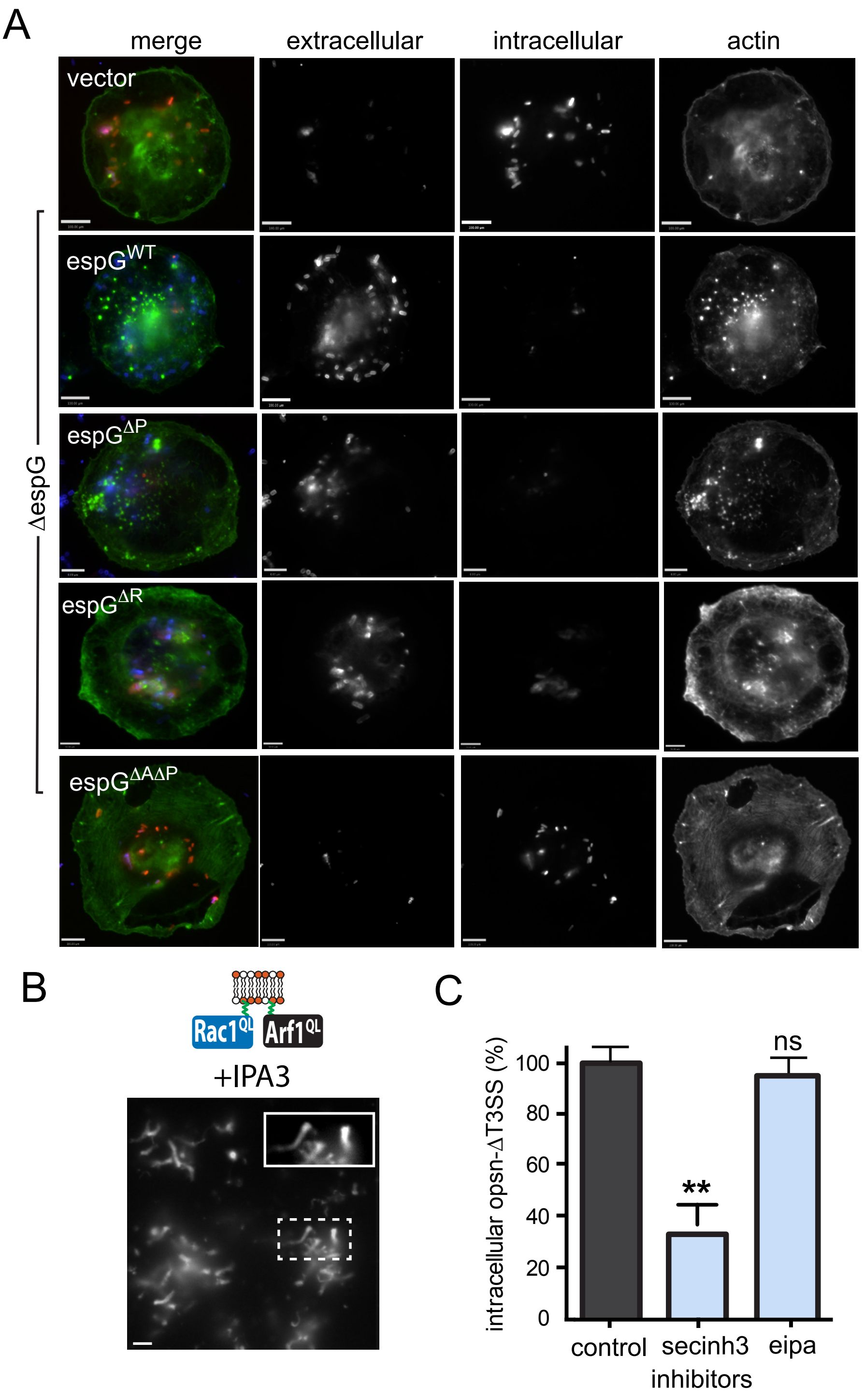

Figure S2. EspG counteracts phagocytosis by binding Arf GTPases (related to Fig 2 and Fig 3).

(**A**) Images showing phagocytosis of EPEC$^{\Delta espG}$ (ΔespG) expressing a control vector or the vector encoding espG variants, namely wild-type (WT), or mutants in binding Rab (EspG$^{\Delta R}$), PAK (EspG$^{\Delta P}$) or both Arf and PAK (EspG$^{\Delta A \Delta P}$). Scale bars 6μm. Quantification shown in Fig 2F. (**B**) WRC-dependent actin-based motility directed by Arf1^{QL} and Rac1^{QL} in extract containing the inhibitor of PAK activation IPA3. (**C**) THP1 phagocytosis of EPEC$^{\Delta T3SS}$ (ΔT3SS) in the presence of opsonising human serum and inhibitors of ARNO (secinh3) or macropinocytosis (eipa). Error bars represent ± SEM. Asterisks indicate a significant difference from control (black bars). Not significant (ns). All experiments in Fig S2 were performed at least three times.

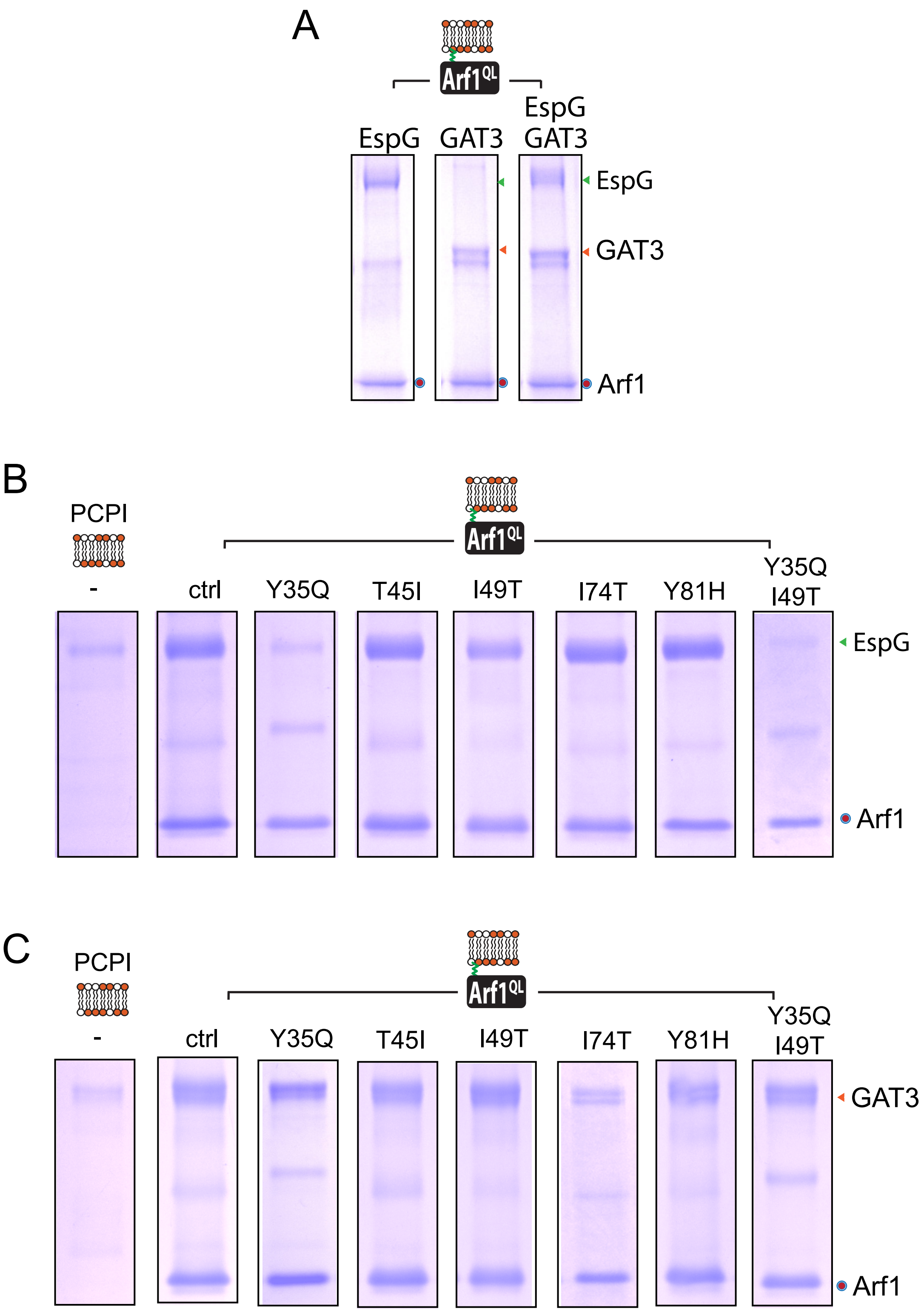

Figure S3. Arf1 interaction with EspG and GAT3 (related to Fig 4).

(**A**) Membrane-anchored Arf1^{QL} interaction with GST-EspG (green arrow) or His-GAT3 (red arrow), or both in combination, in buffer. (**B**) Interaction of GST-EspG with PCPI-membranes alone (-) as a control or with membranes anchored with Arf1^{QL} (ctrl) or Arf1^{QL} derivatives incorporating mutations within the alpha-1 helix (Y35Q), switch 1 (T45I, I49T) or switch 2 (I74T, Y81H) domain as indicated. The results show that EspG binds Arf1 residue Y35 and I49, albeit to a lesser extent (**C**) Experiment performed as (B) with GST-GAT3. The results show that GAT3 weakly interacts with I74. All experiments in Fig S3 were performed at least three times.

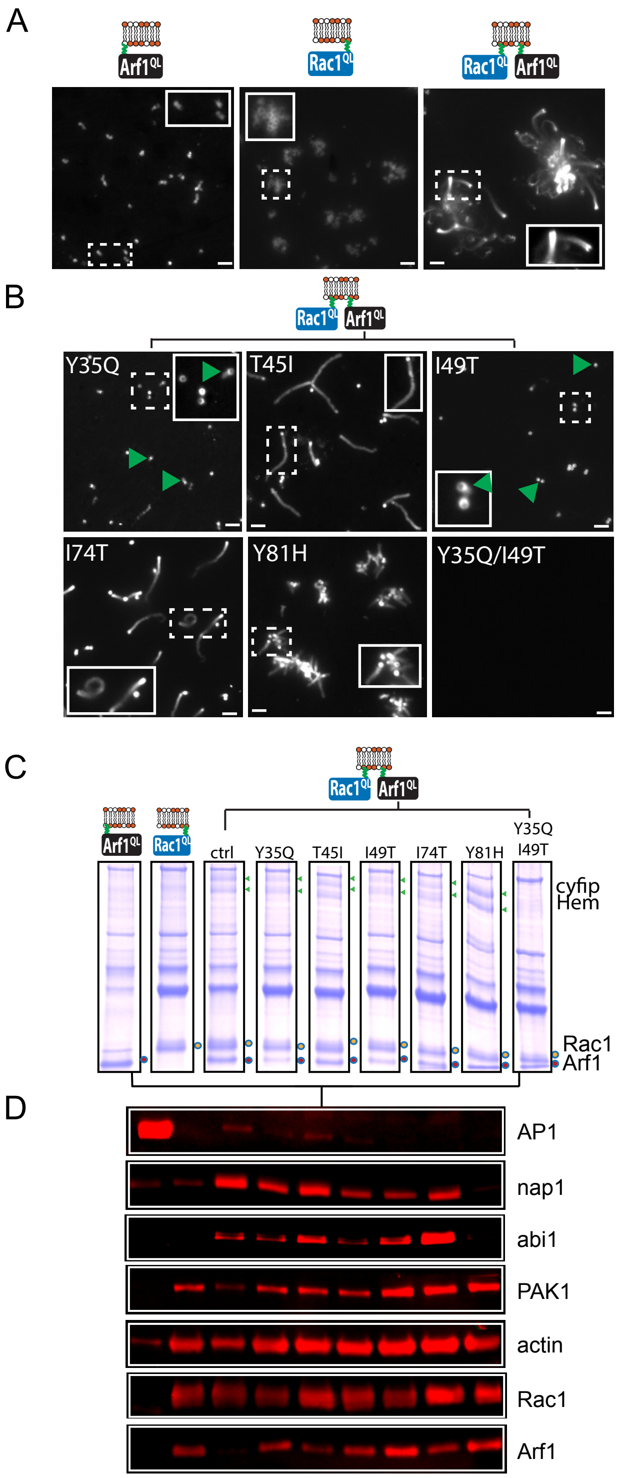

Figure S4. Molecular basis of Arf1 cooperation with Rac1 in WRC recruitment and activation (related to Fig 5).

(**A**) WRC-dependent actin-based motility directed by Arf1^{QL} or Rac1^{QL} alone, or in combination. Insets magnify actin-comet tails. Scale bars 5µm. The results show that only Arf1^{QL} or Rac1^{QL} co-anchored at the membrane activate the WRC (**B**) WRC-dependent actin-based motility directed by Rac1^{QL} in combination with Arf1^{QL} derivatives incorporating mutations within the alpha-1 helix (Y35Q), switch 1 (T45I, I49T) or switch 2 (I74T, Y81H) domain as indicated. Scale bars 5µm. The data show that Arf1 residues Y35 and I49 are required for WRC activation (**C**) Proteins recruited by membrane-anchored Arf1^{QL} or Rac1^{QL} alone, or Rac1^{QL} in combination with Arf1^{QL} containing indicated mutations. Green arrows indicate cyfip and Hem. (**D**) Immunoblotting of samples from (C) with indicated antibodies (right). The data in (C) and (D) show that Arf1 residues Y35 and I49 are required for WRC recruitment (i.e. Y35Q/I49T). All experiments in Fig S4 were performed at least three times.

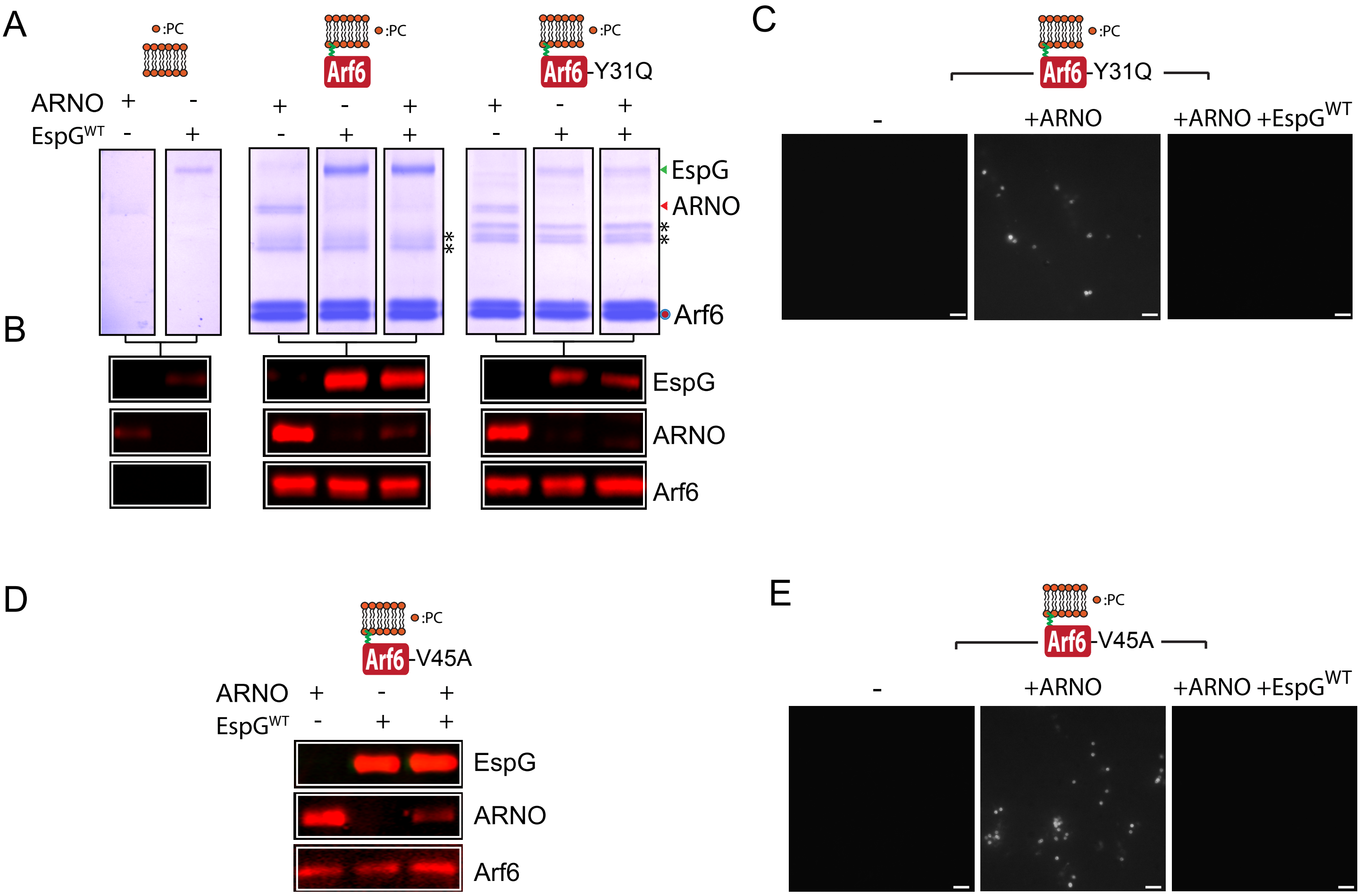

Figure S5. Ability of Arf6 mutants to interact with EspG and ARNO, and activate the WRC (related to the discussion).

(**A**) The interaction of PC membranes alone (left) or membranes anchored with GTPγS-loaded wild-type or Y31Q Arf6 with His-ARNO (red arrow) or GST-EspG (green arrow) alone, or both in combination, in buffer. Asterisks indicate Arf6 dimers. To assess the influence of the Arf6-Y31Q mutation, PC and Arf6 controls shown left are duplicated from Fig 3G for clarity. (**B**) Immunoblotting of samples from (A) with indicated antibodies against GST (EspG), ARNO and Arf6 (right). To assess the influence of the Arf6-Y31Q mutation, PC and Arf6 controls shown left are duplicated from Fig 3G for clarity. (**C**) WRC-dependent actin assembly via membrane-anchored Arf6$^{GTP\gamma S}$-Y31Q (depicted in cartoon) in cell-free extract alone or in extract containing recombinant ARNO in the presence or absence of recombinant EspG^{WT}. Collectively, the results from (A, B and C) show that the Y31Q mutation attenuates interactions with EspG but interactions at the membrane are still sufficient for EspG to impede ARNO-Ar6-Y31Q associations in buffer (A, B) and WRC activation in extract (C). (**D**) Immunoblotting of proteins
