# Supplementary material for: Inhibition of WAVE Regulatory Complex Activation by a Bacterial Virulence Effector Counteracts Pathogen Phagocytosis
Source: Cell Rep. 2016 Oct 11;17(3):697–707. doi: 10.1016/j.celrep.2016.09.039 (PMC5081413; doi:10.1016/j.celrep.2016.09.039)
Supplement: Document S2. Article plus Supplemental Information [file mmc2.pdf]

Cell Reports

Article

Inhibition of WAVE Regulatory Complex Activation by a Bacterial Virulence Effector Counteracts Pathogen Phagocytosis

Graphical Abstract

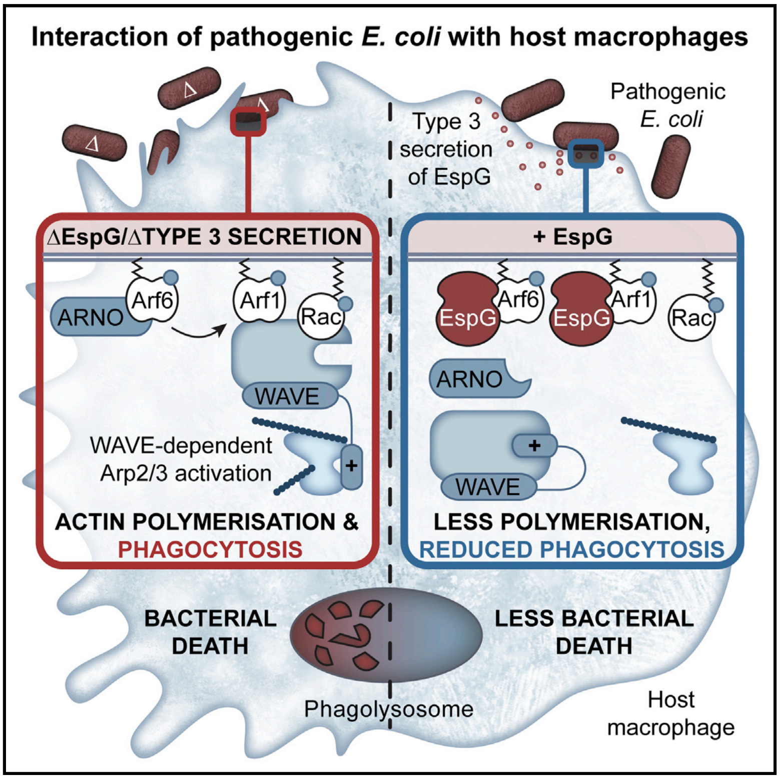

Authors

Daniel Humphreys, Vikash Singh, Vassilis Koronakis

Correspondence

d.humphreys@sheffield.ac.uk (D.H.),
vk103@cam.ac.uk (V.K.)

In Brief

Humphreys et al. find that the virulence effector EspG of enteropathogenic and enterohaemorrhagic *Escherichia coli* inhibits pathogen phagocytosis by inhibiting activation of the WAVE regulatory complex (WRC). EspG uncouples the small GTPases Arf1 and Arf6 from Rac1, thus initiating WRC-dependent actin polymerization.

Highlights

- WAVE regulatory complex (WRC) and Arf and Rac1 direct phagocytosis of EPEC and EHEC
- Virulence effector EspG inhibits the WRC to counteract pathogen phagocytosis
- EspG blocks Arf6 signaling to ARNO that activates Arf1
- EspG blocks Arf1 collaboration with Rac1 and WRC activation

Humphreys et al., 2016, Cell Reports *17*, 697–707
October 11, 2016 Crown Copyright © 2016
http://dx.doi.org/10.1016/j.celrep.2016.09.039

CellPress

OPEN ACCESS
CellPress

Inhibition of WAVE Regulatory Complex Activation by a Bacterial Virulence Effector Counteracts Pathogen Phagocytosis

Daniel Humphreys,[1,*] Vikash Singh,[1] and Vassilis Koronakis[1,2,*]
[1]Department of Pathology, University of Cambridge, Tennis Court Road, Cambridge CB2 1QP, UK
[2]Lead Contact
*Correspondence: d.humphreys@sheffield.ac.uk (D.H.), vk103@cam.ac.uk (V.K.)
http://dx.doi.org/10.1016/j.celrep.2016.09.039

SUMMARY

To establish pathogenicity, bacteria must evade phagocytosis directed by remodeling of the actin cytoskeleton. We show that macrophages facilitate pathogen phagocytosis through actin polymerization mediated by the WAVE regulatory complex (WRC), small GTPases Arf and Rac1, and the Arf1 activator ARNO. To establish extracellular infections, enteropathogenic (EPEC) and enterohaemorrhagic (EHEC) *Escherichia coli* hijack the actin cytoskeleton by injecting virulence effectors into the host cell. Here, we find that the virulence effector EspG counteracts WRC-dependent phagocytosis, enabling EPEC and EHEC to remain extracellular. By reconstituting membrane-associated actin polymerization, we find that EspG disabled WRC activation through two mechanisms: EspG interaction with Arf6 blocked signaling to ARNO while EspG binding of Arf1 impeded collaboration with Rac1, thereby inhibiting WRC recruitment and activation. Investigating the mode of EspG interference revealed sites in Arf1 required for WRC activation and a mechanism facilitating pathogen evasion of innate host defenses.

INTRODUCTION

Professional phagocytes cells represent the first line of host defense against bacterial pathogens. To eradicate pathogenic bacteria, professional phagocytes employ myriad host cell-surface receptors that bind the target bacterium directly (e.g., bacterial surface sugars) or indirectly through host-derived opsonins (e.g., antibodies, complement) (Celli and Finlay, 2002; Sarantis and Grinstein, 2012). Receptor binding triggers polymerization of actin filaments that guide the plasma membrane around the pathogen to facilitate bacterial uptake and destruction within an intracellular microbicidal phagolysosome compartment. The actin polymerization requires Rho GTPases Rac1 and Cdc42 that anchor by lipid prenylation to the membrane where they recruit and activate myriad cellular effectors responsible for directing cytoskeleton remodeling via the Arp2/3 complex (Caron and Hall, 1998; May et al., 2000).

Counteracting phagocytosis is a central paradigm in bacterial pathogenicity. For example, to inhibit opsonin-dependent *trans*-phagocytosis *Staphylococcus aureus* secretes protein A, which sequesters antibodies, while several pathogens use cell-surface capsule polysaccharides to inhibit deposition of complement (Celli and Finlay, 2002; Sarantis and Grinstein, 2012). However, phagocytes offset this strategy through myriad non-opsononic phagocyte receptors that directly bind bacteria and mediate *cis*-phagocytosis independent of opsonins. Nevertheless, uniting the diverse uptake mechanisms is the role of the actin cytoskeleton whose remodeling is required for phagocytosis (May et al., 2000). Consequently, pathogens have evolved sophisticated measures to interfere with the actin cytoskeleton and antagonize a spectrum of phagocytic mechanisms at the molecular level.

Enteropathogenic and enterohaemorrhagic *Escherichia coli* (EPEC and EHEC) are major global human health threats causing gastroenteritis and bloody diarrhea, respectively (Hartland and Leong, 2013). To cause disease, they inject a cocktail of virulence effectors into host cells via a type 3 secretion system (T3SS) to enable cell-surface colonization on intestinal epithelia where the pathogen forms lesions characterized by the destruction of brush border microvillii. Here, the bacteria encounter macrophages that infiltrate sites of infection yet EPEC and EHEC are able to block their own phagocytosis through the injected virulence effectors (Santos and Finlay, 2015). Indeed, mutants of type 3 secretion are phagocytosed by macrophages (Goosney et al., 1999; Marchès et al., 2008). Four virulence effectors are known to contribute to anti-phagocytosis (Santos and Finlay, 2015); EspB interacts with the actin binding motor protein myosin-1c (Iizumi et al., 2007), EspF inhibits PI3 kinase signaling (Celli et al., 2001), EspH inhibits the Dbl subfamily of Rho guanine nucleotide exchange factors (GEFs) (Dong et al., 2010) and EspJ impedes phagocytosis through inhibition of Src kinase activity (Young et al., 2014).

EPEC and EHEC employ multiple mechanisms to disable phagocytosis. While it is clear that the pathogens target the actin cytoskeleton, we do not yet understand the identity of the cellular actin nucleation machinery governing pathogen phagocytosis

 Crown Copyright © 2016
This is an open access article under the CC BY license (http://creativecommons.org/licenses/by/4.0/).

and therefore the mechanisms of bacterial interference. Consequently, we first sought to identify the players underlying the actin filament polymerization that are targeted by virulence effectors.

RESULTS

EPEC Opposes Phagocytosis Directed by the WAVE Regulatory Complex

To address how pathogenic *Escherichia coli* resist engulfment by macrophages, we infected differentiated human THP1 macrophage cells with wild-type EPEC ($EPEC^{WT}$) or T3SS-deficient mutant EPEC ($EPEC\Delta^{T3SS}$) labeled with pHrodo, a pH-sensitive dye that fluoresces red in the low pH of phagosomes and signified pathogen uptake (Figure 1A). Intracellular bacteria were inaccessible to antibodies against EPEC that marked extracellular bacteria. Only ~27% of $EPEC^{WT}$ were found intracellular within phagosomes of host cells (actin) and labeling of extracellular bacteria with antibodies demonstrated that the majority of bacteria had counteracted phagocytosis (Figures 1A and 1B). In contrast, very few extracellular $EPEC\Delta^{T3SS}$ were observed as ~92% of bacteria had been phagocytosed. Equivalent results were observed in RAW267.4 mouse macrophage cells (Figure S1A), confirming that EPEC fights phagocytosis using T3SS effectors.

Given the central role of Arp2/3-dependent actin polymerization in phagocytosis (May et al., 2000), it seemed likely that EPEC targeted activators of Arp2/3. The WAVE regulatory complex (WRC) is one such activator that is known to drive pathogen macropinocytosis in epithelial cells (Humphreys et al., 2012b, 2013) and has been implicated in phagocytosis by *Dictyostelium* (Seastone et al., 2001), mammalian granulocytes (Pils et al., 2012), and neutrophils and macrophages (Park et al., 2008). The WRC is a heteropentameric complex comprising Cyfip, Nap/Hem, Abi, and HSPC300 or their homologs (Gautreau et al., 2004), which must be activated directly by Rac1 (Miki et al., 1998) in combination with additional potentiating signals including that mediated directly by Arf1 GTPase (Koronakis et al., 2011; Krause and Gautreau, 2014). Though whether Rac1 and Arf1 co-operate in WRC-mediated phagocytosis is not known.

To address whether EPEC effectors resist WRC-dependent phagocytosis, THP1 macrophages were depleted of the WRC component Hem by siRNA knockdown before infection with $EPEC\Delta^{T3SS}$ (Figures 1C, S1B, and S1C). In contrast to mock knockdown cells where ~84% of $EPEC\Delta^{T3SS}$ were phagocytosed (Figures 1C, S1B, and S1C), pathogen uptake was significantly impaired in Hem knockdown cells where it was reduced to ~33% with the majority of bacteria observed extracellularly (Figures 1C and S1C). Little is known about WRC regulation in macrophages, and we sought to address the significance of co-operating small GTPases Arf1 and Rac1. Here, knockdown of WRC activators Arf1 and Rac1 also reduced $EPEC\Delta^{T3SS}$ uptake to ~38% and ~35%, respectively (Figure 1C), which enabled bacteria to remain on the cell surface (Figure S1C) and established that EPEC effectors oppose WRC-dependent phagocytosis regulated by cooperating small GTPases Arf1 and Rac1.

EPEC Effector EspG Disables WAVE Regulatory Complex Activation

Given the prominent role of small GTPases in $EPEC\Delta^{T3SS}$ phagocytosis, we reasoned that EPEC effectors might directly target these players to resist uptake. This strategy is a central virulence strategy employed by many pathogens that deactivate small GTPases through effectors possessing GTPase-activating activity that promote GTP hydrolysis (Dean, 2011). Even so, EPEC encodes no known GTPase-activating protein (GAP) of Rac1 or Arf1 and therefore likely uses an alternative mechanism. One possibility included the EPEC effector EspG that was previously shown to act as a molecular scaffold by simultaneously binding the Rac1 effector p21 activated kinase (PAK) and GTP-bound Arf GTPases (e.g., Arf1, Arf6) (Selyunin et al., 2011). EspG directly activates PAK, while EspG interaction with Arf sterically hinders Arf GAPs, thereby maintaining the GTP-bound form of Arf, which still has portions of its switch 1 and 2 domains exposed to permit interactions with some, but not all, of its cellular effectors (Selyunin et al., 2011, 2014).

First, we examined whether EspG antagonizes WRC-dependent cytoskeleton remodeling by infecting THP1 macrophages with pHrodo-labeled $EPEC^{WT}$ or an isogenic strain with null mutations in espG and its close homolog espG2, henceforth $EPEC\Delta^{espG}$ (Figure 1D). In contrast to wild-type bacteria, $EPEC\Delta^{espG}$ bacteria were incapable of resisting phagocytosis, and ~85% were observed intracellularly mirroring the phagocytosis of $EPEC\Delta^{T3SS}$ (Figures 1B and 1D). Furthermore, this EspG role appears conserved as ΔespG strain of the related pathogen EHEC was also susceptible to phagocytic uptake while wild-type EHEC were resistant (Figure S1D).

We next investigated the mechanism of WRC interference by EspG. The phosphoinositide PIP3 is known to activate the WRC through Rac1 and Arf GTPases (Lebensohn and Kirschner, 2009; Koronakis et al., 2011), and PIP3 is a major regulator of pathogen phagocytosis (Cox et al., 1999; Celli et al., 2001; Quitard et al., 2006), but how EPEC counteracts PIP3-driven pathways is unclear. We first reconstituted WRC-dependent actin polymerization driven by PIP3 using a motility assay in cell-free extracts as previously described (Hume et al., 2014). Silica microspheres coated with a phospholipid bilayer containing equal amounts of phosphatidylcholine (PC) and phosphatidylinositol (PI) plus 2% PIP3 (PIP3) were added to cell-free extract containing fluorescent rhodamine-labeled actin and non-hydrolysable GTPγS to activate GTPases. In the control, PIP3 microspheres triggered actin polymerization and generated actin-comet tails (of ~14 μm) on the membrane surface that propelled the beads through the extract (Figure 1E). When PIP3-driven motility was examined in extract containing purified recombinant EspG, actin comet tail formation was abrogated and there was no actin assembly on the membrane surface (+EspG). This mirrored the phenotype observed with a Rac1 inhibitor (EHT1864) (Figure 1E) indicating EspG inhibition of the WRC.

To demonstrate that EspG was blocking WRC activation by Arf1 and Rac1, PC:PI microspheres (i.e., without PIP3) were anchored with purified constitutively active GTP-bound myristoylated Arf1-Q71L ($Arf1^{QL}$) and prenylated Rac1-Q61L ($Rac1^{QL}$) and then added to cell-free extract in the presence or absence of

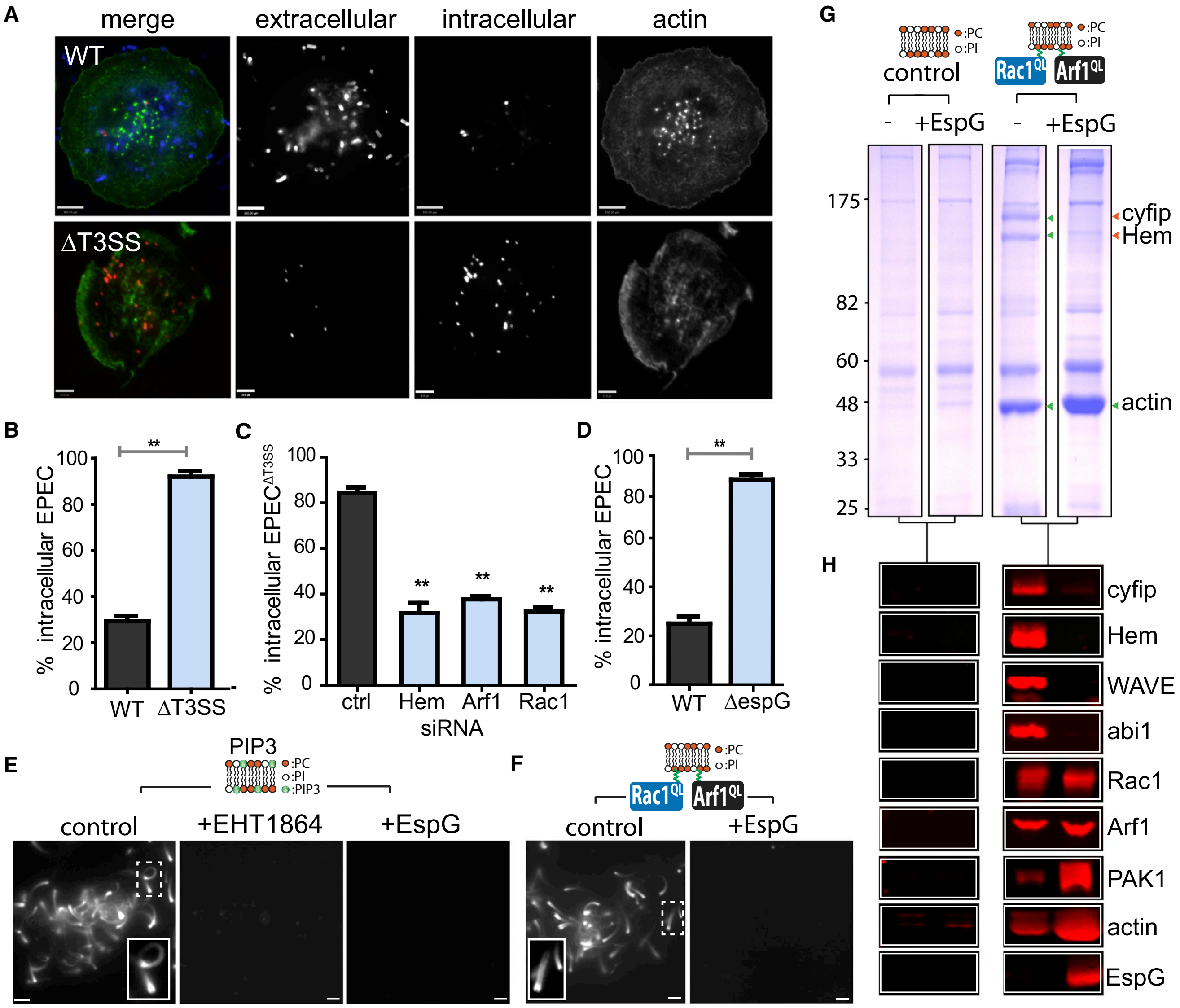

Figure 1. EspG Interference of the WAVE Regulatory Complex

(A) Phagocytosis of pH-Rodo-labeled wild-type and ΔT3SS EPEC. Merged images show intracellular bacteria (red), extracellular bacteria (blue), and host cells (actin). Grayscale shown for clarity. Scale bars, 6 μm.

(B) Bar chart quantifying phagocytosis from experiment in (A).

(C) Phagocytosis of EPECΔ^{T3SS} (ΔT3SS) in THP1s transfected with Hem, Rac1, or Arf1 siRNA.

(D) Phagocytosis of EPECΔ^{espG} (ΔespG). Asterisks indicate a significant difference from control (black bars).

(E) Actin-based motility of PIP3-containing microspheres (depicted in cartoon) in cell-free extract containing a Rac1 inhibitor (+EHT1864) or EspG. Insets magnify actin-comet tails. Scale bars, 5 μm.

(F) WRC-dependent actin-based motility of PC:PI phospholipid bilayers microspheres co-anchored with Arf1^{QL} and Rac1^{QL} in cell-free extract containing fluorescent rhodamine-actin in the presence (+EspG) or absence (control) of recombinant EspG.

(G) Proteins recruited by PC:PI-coated microspheres alone (control) or co-anchored with Arf1^{QL} and Rac1^{QL} (colored circles) from extract (–/+ EspG) were analyzed by SDS-PAGE and Coomassie blue staining. Green arrows indicate cyfip, Hem, or actin. Orange arrows indicate absence of cyfip and Hem. Molecular weight markers in kilodaltons (left).

(H) Immunoblotting of samples from (G) with indicated antibodies (right).

In bar charts (B)–(D), error bars represent ± SEM. See also Figure S1.

EspG (Figure 1F). WRC-dependent actin comet tail formation (~15 μm) was observed in extracts containing PC:PI microspheres co-anchored with Arf1^{QL} and Rac1^{QL}, but actin polymerization was abolished in extract containing EspG.

To further examine how EspG disables WRC-dependent actin polymerization, we scaled up the motility assays to isolate the components recruited to the membrane co-anchored with Arf1^{QL} and Rac1^{QL} in the presence or absence of EspG (Figures

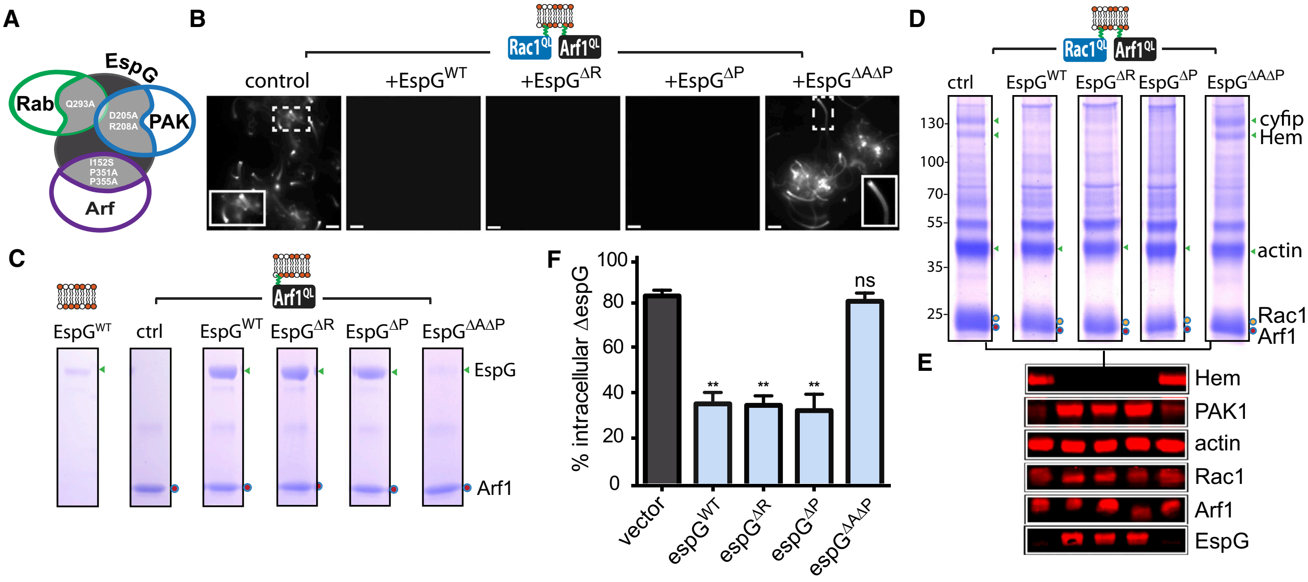

Figure 2. EspG Interaction with Arf GTPase Blocks the WRC

(A) Cartoon depicting key residues (white) in EspG (black) responsible for interaction with Rabs (green), PAK (blue), or Arf (purple).

(B) WRC-dependent actin-based motility via membrane-anchored Arf1^{QL} and Rac1^{QL} (depicted in cartoon) alone (control) or in the presence of EspG, namely, wild-type (WT) or mutants in binding Rab (EspGΔ^{R}), PAK (EspGΔ^{P}) or both Arf and PAK (EspGΔ^{A}Δ^{P}).

(C) Interaction of EspG derivatives with membrane-anchored Arf1^{QL} in buffer.

(D) Proteins recruited by membrane-anchored Arf1^{QL} and Rac1^{QL} (colored circles) in the presence or absence of EspG derivatives as (B) analyzed by SDS-PAGE. Molecular weight markers in kilodaltons (left).

(E) Immunoblotting of samples from (D) with indicated antibodies (right).

(F) Phagocytosis of EPECΔ^{espG} (ΔespG) expressing a control vector or the vector encoding espG variants described in (B).

Error bars represent ± SEM. Asterisks indicate a significant difference from control (black bars). ns, not significant. See also Figure S2.

1G and 1H). PC:PI microspheres alone (control) or co-anchored with Arf1^{QL} and Rac1^{QL} were each incubated in cell-free extract with or without recombinant EspG before being isolated and extensively washed then recruited proteins analyzed by SDS-PAGE and immunoblotting (Figures 1G and 1H). Control microspheres (–) only recruited non-specific proteins, which was also the case when EspG was present (Figures 1G and 1H). In contrast, microspheres co-anchored with Arf1^{QL} and Rac1^{QL} recruited the WRC components cyfip and Hem (Figure 1G, green arrows). Their recruitment was confirmed by immunoblotting (Figure 1H), which also verified the presence of WRC components WAVE and abi1, small GTPases Arf1 and Rac1, and actin derived from comet tails. In contrast, WRC recruitment was impeded (orange arrows) by EspG that was found localized at the membrane, which was dependent upon Arf1^{QL} and Rac1^{QL} (Figures 1G and 1H). Thus, EspG impedes WRC recruitment and activation, PIP3-mediated actin filament polymerization, and WRC-dependent phagocytosis of EPEC and EHEC.

EspG Targeting of Arf1 Antagonizes WRC-Mediated Phagocytosis

EspG is a multifunctional virulence effector and may interfere with WRC-mediated cytoskeleton remodeling in several ways: EspG binds active Arf GTPases and deactivates Rab GTPases (Selyunin et al., 2011; Dong et al., 2012), and both Arf and Rab GTPases are known to promote Rac1-dependent actin filament polymerization (Palamidessi et al., 2008; Koronakis et al., 2011). EspG also activates PAK (Selyunin et al., 2011) that modulates actin filament dynamics, e.g., by deactivating cofilin (Edwards et al., 1999). Consistent with a possible role for PAK1, the presence of EspG at membranes co-anchored with Arf1^{QL} and Rac1^{QL} was co-incident with enhanced PAK1 recruitment (Figure 1H).

To resolve how EspG disables WAVE complex activation, we first purified recombinant EspG mutants (Figure 2A) incapable of deactivating Rabs (EspGΔ^{R}; mutation Q293A) (Dong et al., 2012), binding PAK (EspGΔ^{P}; D205A, R208A) (Germane and Spiller, 2011), or binding Arf and PAK (EspGΔ^{A}Δ^{P}; I152S, P351A, P355A) (Selyunin et al., 2011) before assessing interference with WRC-dependent actin-based motility directed by Arf1^{QL} and Rac1^{QL} (Figure 2B). Like EspG^{WT}, mutant derivatives EspGΔ^{R} and EspGΔ^{P} both abrogated actin-comet tail formation showing that EspG interaction with PAK and its Rab GAP activity were dispensable for WRC inhibition. This was not the case for EspGΔ^{A}Δ^{P}, which had no effect on WRC activity as actin comet tails (of ~14 μm as control) were formed and the microspheres moved through the extract. Thus, EspG targeting of Arf1 and not PAK or Rabs blocked WRC activation.

Consistent with this view, only EspGΔ^{A}Δ^{P} was deficient in binding Arf1^{QL}-anchored membranes in buffer (Figure 2C). Moreover, when EspG^{WT} was pre-incubated with membranes co-anchored with Arf1^{QL} and Rac1^{QL} to form an Arf1-EspG

complex before incubation in extract, WRC-dependent actin comet tail formation was not observed (data not shown). Furthermore, chemical inhibitors of PAK (i.e., IPA3) have been shown to inhibit activation by EspG (Selyunin et al., 2011), yet WRC-dependent actin-based motility was observed in PAK-inhibited extract (Figure S2B), reaffirming that EspG inhibits the WRC independently of PAK.

To further examine EspG inhibition of the WRC, we assessed WRC recruitment by $Arf1^{QL}$ and $Rac1^{QL}$ from cell extract in the presence of the EspG derivatives (Figures 2D and 2E). WRC recruitment to the membrane was obstructed by $EspG^{WT}$, $EspG\Delta^{R}$, and $EspG\Delta^{P}$ but not $EspG\Delta^{A}\Delta^{P}$ as exemplified by the SDS-PAGE (Figure 2D, green arrows) and immunoblotting of Hem (Figure 2E). All EspG variants except $EspG\Delta^{A}\Delta^{P}$ were recruited to $Arf1^{QL}$ and $Rac1^{QL}$ co-anchored membranes. Interestingly, $EspG\Delta^{P}$ but not $EspG\Delta^{A}\Delta^{P}$ recruited PAK1 indicating that PAK recruitment was dependent upon the Arf1-EspG interaction and localization of the virulence effector at the membrane.

In parallel, we assessed phagocytic uptake of $EPEC\Delta^{espG}$ expressing either $EspG^{WT}$, $EspG\Delta^{R}$, $EspG\Delta^{P}$, $EspG\Delta^{A}\Delta^{P}$, or the empty vector as a control (Figures 2F and S2A). THP1 macrophages phagocytosed ~82% of $EPEC\Delta^{espG}$ encoding the vector alone, while bacteria expressing $EspG^{WT}$ resisted WRC-dependent uptake that was reduced to ~35% (Figures 2F and S2A), mirroring the resistance imposed by wild-type EPEC (Figure 1). Similarly, $EPEC\Delta^{espG}$ expressing $EspG\Delta^{R}$ or $EspG\Delta^{P}$ also antagonized phagocytosis. In contrast, ~79% of bacteria expressing $EspG\Delta^{A}\Delta^{P}$ were incapable of resisting the WRC and were phagocytosed to the same extent as the $EPEC\Delta^{espG}$ vector control strain. Thus, EspG-mediated interaction with Arf GTPases, and not PAK or Rabs, combats WRC-directed pathogen phagocytosis.

Next, we examined whether EspG could counteract the activities of other pathogens dependent upon the WRC. In contrast to extracellular pathogens EPEC and EHEC, *Salmonella* Typhimurium is an intracellular pathogen that invades host epithelial cells by activating the WRC-Rac1-Arf1 axis (Humphreys et al., 2012b, 2013). When we examined *Salmonella* invasion in HeLa cells expressing HA-tagged EspG, pathogen uptake was reduced by ~64% relative to control, which was not observed in cells expressing $EspG\Delta^{A}\Delta^{P}$ (Figure 3A), thus providing further evidence of EspG interference of WRC-dependent cytoskeleton remodeling.

EspG Incapacitates Arf6 and ARNO Upstream of WRC Activation

EspG is known to bind Arf6 in an analogous fashion to Arf1 (Selyunin et al., 2011), though no function has been ascribed for this host-pathogen interaction. To trigger WRC-dependent invasion, *Salmonella* Typhimurium hijacks Arf6 to recruit and activate the Arf1 GEF ARNO of the cytohesisn family (Humphreys et al., 2013; Stalder et al., 2011). We therefore speculated that EspG might also incapacitate WRC activation by inhibiting Arf6 upstream of ARNO-mediated activation of Arf1.

First, we examined whether the cytohesin family (i.e., ARNO) facilitated phagocytosis. THP1 cells treated with the cytohesin inhibitor secinh3 impeded the relative uptake of $EPEC\Delta^{espG}$ from ~82% to ~30% (Figures 3B and 3C). ARNO is known to facilitate macropinocytosis (Humphreys et al., 2012b, 2013), but the uptake of $EPEC\Delta^{espG}$ was not affected by the macropinocytosis inhibitor eipa (Figure 3B). Secinh3 but not eipa also inhibited the phagocytosis of $EPEC\Delta^{espG}$ opsonized with human serum (Figure S2C). Thus, ARNO directs phagocytosis of EPEC, which was counteracted by EspG.

We next examined the mechanism by which EspG antagonized ARNO by reconstituting Arf6-driven activation of WRC as previously reported (Humphreys et al., 2013). PC:PI microspheres anchored with recombinant myristoylated Arf6 activated with GTPγS were incubated in extract with or without (–) recombinant ARNO (Figure 3D). Arf6 only triggered actin assembly in the presence of ARNO, but this was abrogated in extract containing $EspG^{WT}$ but not $EspG\Delta^{A}\Delta^{P}$. To determine how EspG impeded Arf6-dependent actin polymerization, PC:PI microspheres were isolated from extract then analyzed by immunoblotting (Figure 3E). To trigger WRC activation, Arf6 must recruit ARNO (Humphreys et al., 2013). Indeed, Arf6 alone (– ARNO) recruited very little Arf1 and Hem, which was enhanced upon addition of recombinant ARNO (+ ARNO). However, in the presence of EspG, Arf6 was incapacitated as the recruitment of ARNO as well as downstream players Arf1 and Hem were impeded. This was not the case with the Arf binding mutant $EspG\Delta^{A}\Delta^{P}$ that had no effect on the Arf6 cascade.

Finally, to investigate whether EspG directly inhibits ARNO via binding to Arf6, we examined interactions in buffer with purified components and PC-coated microspheres (Figures 3F and 3G) that minimize known ionic interactions between the ARNO pleckstrin-homology domain and acidic phospholipids such as PI (Macia et al., 2000). ARNO weakly bound PC microspheres alone, but its recruitment was potentiated by Arf6 (Figures 3F and 3G). In the presence of $EspG^{WT}$, the virulence effector was recruited through Arf6 that blocked interaction with ARNO (Figures 3F and 3G). In contrast, the Arf binding mutant $EspG\Delta^{A}\Delta^{P}$ was not recruited by Arf6 and was incapable of impeding ARNO (Figures 3F and 3G). Thus, EspG directly disables Arf6-dependent actin polymerization by blocking signaling to its cellular effector ARNO.

The Molecular Basis of WRC Interference by EspG

Activated Arf1 mediates interaction with cellular effectors via its switch 1 (residues 40–51) and 2 (68–81) domains (Nie et al., 2003). EspG exhibits an unusual Arf binding interface that is rotated away from the switch 2 site (Figure 4A) where it interacts with the switch 1 and the alpha-1 helix (29-37) positioned outside of the canonical switch regions (Selyunin et al., 2011). Consequently, Arf1 bound to EspG can still bind cellular effectors that interact with its switch 2 domain such as the Arf binding GAT domain of GGA vesicle adaptors (Selyunin et al., 2014; Kuai et al., 2000) as depicted in Figure 4A and confirmed experimentally in Figure S3A.

We took advantage of the distinct binding modes of EspG and GAT3 to investigate the mechanism of Arf1-mediated WRC activation and EspG interference. First, we examined actin-based motility in extracts containing equivalent concentrations of either EspG or GAT3 (Figure 4B). EspG was more potent at inhibiting WRC than GAT3, which impeded robust comet tail formation but still permitted initiation of actin assembly and small comet

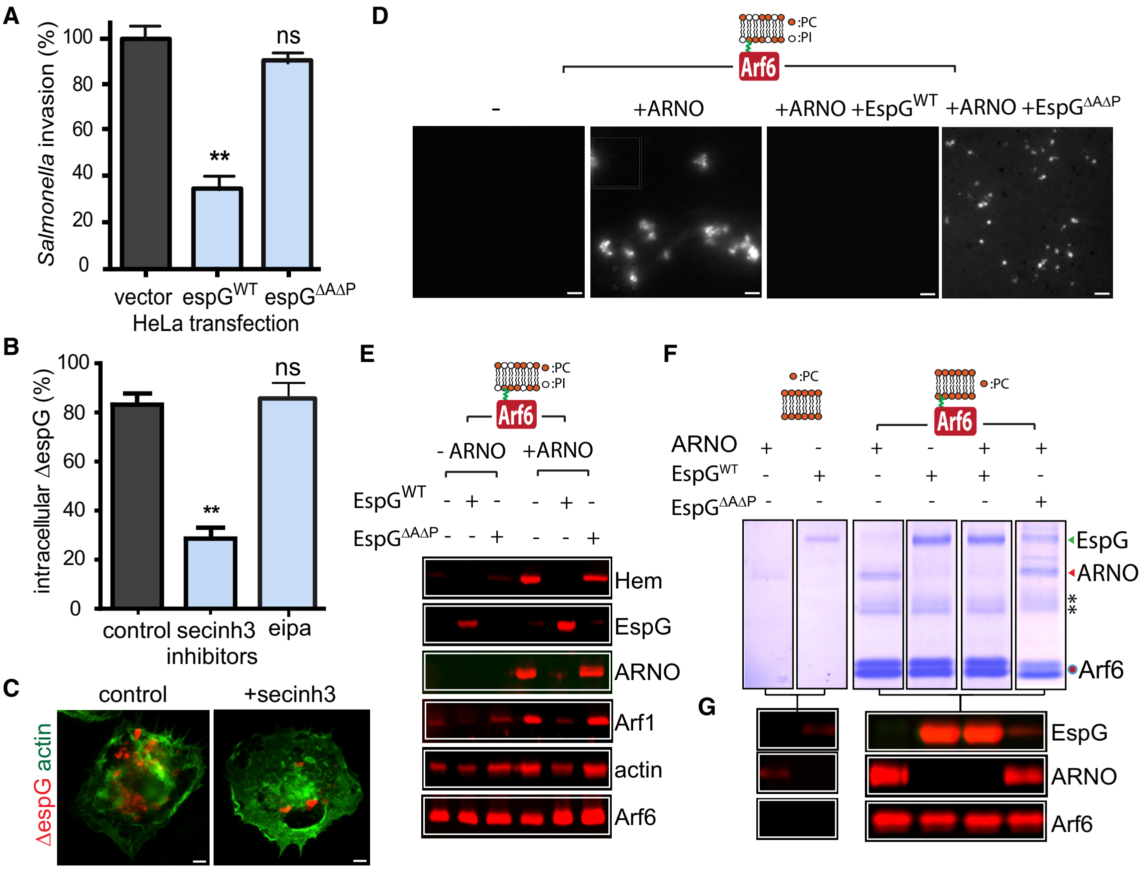

Figure 3. EspG Incapacitation of Arf6 Signaling to ARNO and the WRC

(A) *Salmonella* invasion by macropinocytosis into non-phagocytic HeLa cells expressing a control vector or the vector encoding wild-type EspG (espG^{WT}) or a mutant in binding both Arf and PAK (espGΔ^{A}Δ^{P}). Values for invasion were normalized to the control

(B) THP1 phagocytosis of EPECΔ^{espG} (ΔespG) in the presence of inhibitors of ARNO (secinh3) or macropinocytosis (eipa). Asterisks indicate a significant difference from control (black bars). Not significant (ns). See also Figure S3C.

(C) Imaging of phagocytosed EPECΔ^{espG} (ΔespG) bacteria (red) and THP1 actin cytoskeleton (green) from experiment in (B). Scale bars, 4 μm.

(D) WRC-dependent actin assembly via membrane-anchored Arf6$^{GTP\gamma S}$ (depicted in cartoon) in cell-free extract alone or in extract containing recombinant ARNO in the presence or absence of recombinant EspG derivatives described in (A).

(E) Immunoblotting of proteins recruited from cell extract by phosphatidylcholine:phosphatidylinositol (PCPI) membranes anchored with Arf6$^{GTP\gamma S}$ in the presence of ARNO and EspG derivatives described in (A).

(F) The interaction of PC membranes alone (left) or membranes anchored with GTPγS-loaded Arf6 with His-ARNO (red arrow) or GST-EspG (green arrow) alone, or both in combination, in buffer. Asterisks indicate Arf6 dimers.

(G) Immunoblotting of samples from (A) with indicated antibodies against GST (EspG), ARNO, and Arf6 (right).

In bar charts (A) and (B), error bars represent ± SEM.

tails (magnified inset) of ~8 μm relative to ~14 μm observed with the control. Indeed, while Arf1^{QL} and Rac1^{QL} recruited both EspG and GAT3 to the membrane (Figure 4C, GST), EspG but not GAT3 impeded WRC recruitment (Figure 4C; Hem, Figure S3B, green arrows). It is not known how Arf1 activates the WRC but the results suggested that EspG inhibits recruitment of Arf1 effectors that bind switch 1. This hypothesis was further substantiated by immunoblotting of the switch 1-binding protein AP-1 (Austin et al., 2002; Ren et al., 2013) that was recruited in the presence of GAT3 but not EspG (Figure 4C, AP-1).

EspG interaction with Arf1 is key to inhibiting WRC activation (Figure 2). Thus, we set out to resolve the molecular basis of EspG interference further by purifying an array of Arf1^{QL} derivatives incorporating mutations within the alpha-1 helix (Y35Q), switch 1 (T45I, I49T), or switch 2 (I74T, Y81H) domain (Figure 4A), which have been implicated in interactions with EspG or its cellular effectors (Selyunin et al., 2011; Kuai et al., 2000). We examined EspG and GAT3 interactions with PC:PI microspheres anchored with each Arf1^{QL} mutant derivative in buffer (Figures 3D and 3E, and comprehensively shown in Figures S3B and S3C). EspG bound control Arf1^{QL} but interaction with anchored Arf1^{QL}-Y35Q was completely abolished and though the effector was still recruited by Arf1^{QL}-I49T, the interaction was evidently weaker (Figure 3D). The remaining Arf1 mutations had no effect. In contrast, GAT3 bound each Arf1^{QL} variant equivalently except the switch 2 mutant Arf1^{QL}-I74T (Figure 3E).

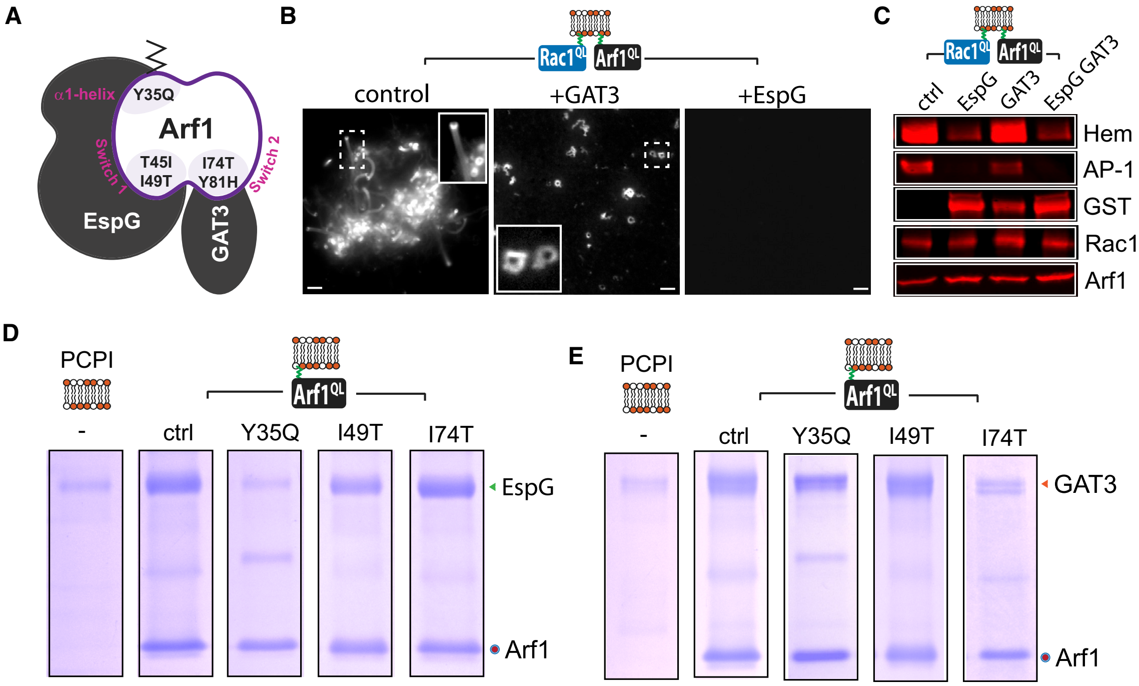

Figure 4. Molecular Basis of WRC Interference by EspG

(A) Cartoon depicting Arf1 interaction with EspG or GAT3 of GGA3 with key residues and domains in Arf1 shown.

(B) WRC-dependent actin-based motility directed by Arf1^{QL} and Rac1^{QL} in extract alone (control), or with GAT3 or EspG. Insets magnify actin-comet tails. Scale bars 5 μm.

(C) Immunoblotting of proteins recruited by membrane-anchored Arf1^{QL} and Rac1^{QL} from extract alone (ctrl), or from extract containing EspG or GAT3, or both in combination. Anti-GST antibodies detected GAT3 and EspG.

(D) Interaction of EspG with PCPI membranes alone (–) or with membranes anchored with Arf1^{QL} (ctrl) or Arf1^{QL} derivatives containing indicated mutations.

(E) Experiment performed as (B) with GAT3.

See also Figure S3.

EspG Targets Arf1 Residues Essential to Cooperation with Rac1 and WRC Activation

As the Arf1 residue Y35, and to a lesser extent I49, likely underlie EspG inhibition of the Arf1-Rac1-WRC axis, we next examined whether these sites in Arf1 were key for collaboration with Rac1 in WRC recruitment and activation at the membrane. In contrast to Arf1^{QL} or Rac1^{QL} alone, only membranes co-anchored with both GTPases (ctrl) triggered recruitment of the WRC (Figures 5A and 5B) and robust actin comet tail formation (Figures 5C and S4A) demonstrating that small GTPase cooperation was required for WRC activation. Interestingly, Arf1^{QL} alone recruited AP-1 (Figure 5B), a marker for classical Arf1 effectors that binds the alpha-1 helix and switch 1 domain of Arf1 (Austin et al., 2002; Ren et al., 2013). However, when Arf1^{QL} combined with Rac1^{QL} (ctrl) the presence of AP-1 was diminished relative to Arf1^{QL} alone, while WRC recruitment was enhanced uncovering a remarkable switch in effector interplay by Arf1 when collaborating with Rac1 (Figure 5B). Thus, when working in synergy with Rac1 the results indicate that Arf1 recruits and activates the WRC via its alpha-1 helix and switch 1 domain in place of classical effectors such as AP-1.

When we examined the Arf1^{QL} derivatives mutated in the alpha-1 helix, switch-1 or switch-2 domain, they all collaborated with Rac1 by recruiting the WRC (Figures 5A and 5B and comprehensively shown in Figures S4C and S4D). Given this observation, we were surprised to find that certain Arf1 mutations had a substantial impairment in WRC activation (Figures 5C and S4B). Like Arf1^{QL}, mutants Arf1^{QL}-T45I, -I74T, and -Y81H formed robust actin comet tails (exemplified by I74Tin Figure 5C). In contrast, the motility of membranes anchored with Arf1^{QL}-Y35Q or -I49T was markedly impaired (Figure 5C). We noticed that a small proportion of actin shells (~10%) surrounding the Arf1^{QL}-Y35Q and Arf1^{QL}-I49T membranes broke symmetry to form stumpy comet tails of ~2 μm (exemplified by green arrows in Figure 5C), indicating weak activation of the WRC. We speculated that the weak activation of the WRC by Arf1^{QL}-Y35Q would be resistant to interference by EspG, which binds Y35 (Figure 4D). Sure enough, while EspG blocked the formation of comet tails generated by the switch 2 mutant Arf1^{QL}-I74T, stumpy comets were still formed by Arf1^{QL}-Y35Q even in the presence of EspG (Figure 5C).

Finally, as both Arf1 residues Y35 and I49 mediated interaction with EspG (Figure 4D), we examined WRC recruitment and actin-based motility at membranes anchored with a double mutant (Figures 5A–5C). Arf1^{QL}-Y35Q/I49T was incapable of collaborating with Rac1^{QL} as the WRC was neither recruited (Figures 5A and 5B) nor activated (Figure 5C). Thus, EspG targets specific residues in the alpha-1 helix and switch 1 domain of Arf1 that

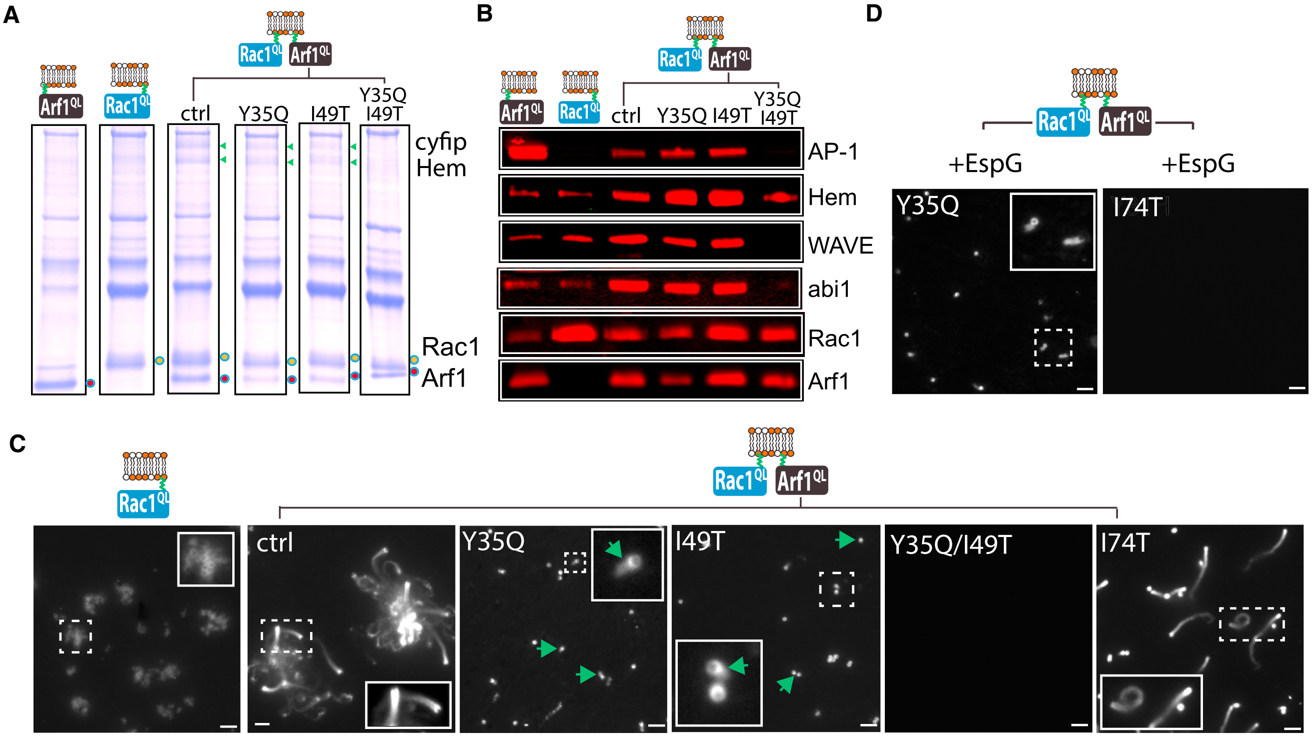

Figure 5. EspG Targets Arf1 Residues Essential to Synergy with Rac1 and WRC Activation

(A) WRC-dependent actin-based motility directed by Rac1^{QL} alone (–) or in combination with Arf1^{QL} (ctrl) or in combination with Arf1^{QL} containing indicated mutations. Insets magnify actin-comet tails. Scale bars, 5 μm.

(B) Proteins recruited by membrane-anchored Arf1^{QL} or Rac1^{QL} alone or Rac1^{QL} in combination with Arf1^{QL} containing indicated mutations as (A). Green arrows indicate cyfip and Hem.

(C) Immunoblotting of samples from (B) with indicated antibodies (right).

(D) WRC-dependent actin-based motility directed by Rac1^{QL} in combination with indicated Arf1^{QL} mutants in extract containing EspG. Insets magnify actin-comet tails. Scale bars, 5 μm.

See also Figure S4.

facilitate small GTPase co-operation and actin filament polymerization by the WRC.

DISCUSSION

To avoid phagocytosis bacterial pathogens employ a wide range of strategies. For example, many pathogens secrete immunoglobulin proteases to cleave antibodies and impede FcR-mediated uptake (Sarantis and Grinstein, 2012). However, not all phagocytic mechanisms are driven by opsonization emphasizing the need for other inventive virulence strategies. We show that EPEC and EHEC circumnavigate this problem by inhibiting WRC signaling to the actin cytoskeleton whose remodeling is at the very center of phagocytosis. The role of Rac1 and Arf6 in phagocytosis is well established (Niedergang et al., 2003; Zhang et al., 1998; Caron and Hall, 1998), yet the contribution of the WRC (Park et al., 2008) and Arf1 is less clear (Beemiller et al., 2006; Sendide et al., 2005). WRC activation by Arf1 and Rac1 is known to mediate lamellipodia formation and *Salmonella* macropinocytosis into host cells (Humphreys et al., 2012a, 2013). Here, we show a crucial role for the WRC in pathogen phagocytosis and establish that collaborating WRC activators Arf1 and Rac1 are required. Structural homologs cyfip and Hem of WRC are thought to oppose the plasma membrane where small GTPases are anchored (Chen et al., 2010). As Rac1 is known to bind cyfip (Kobayashi et al., 1998), it is possible that Arf1 collaborates with Rac1 by binding Hem (depicted in Figure 6) or through an unidentified Arf1 effector acting as an intermediate. By using EspG to probe the relationship between Arf1 and Rac1, we identify key residues in the Arf1 alpha-1 helix (Y31) and switch-1 domain (I49) that underpin its collaboration with Rac1 in WRC recruitment and activation at the membrane. Interestingly, we found that the equivalent residues in Arf6 (Y31 and V45) were dispensable for Arf6 activation of ARNO (Figure S5), and, although the EspG interactions with Arf6-Y31Q were attenuated as previously reported (Selyunin et al., 2011), the interactions at the membrane were sufficient to block ARNO-dependent signaling to the WRC mediated by either Arf6-Y31Q or Arf6-V45A (Figure S5). Thus, the sites on Arf6 that permit signaling to ARNO and interference by EspG are distinct from those in Arf1 that cooperate with Rac1 in WRC signaling at the membrane, and perhaps reflect differences in the primary sequence of Arf1 and Arf6 (70% identity).

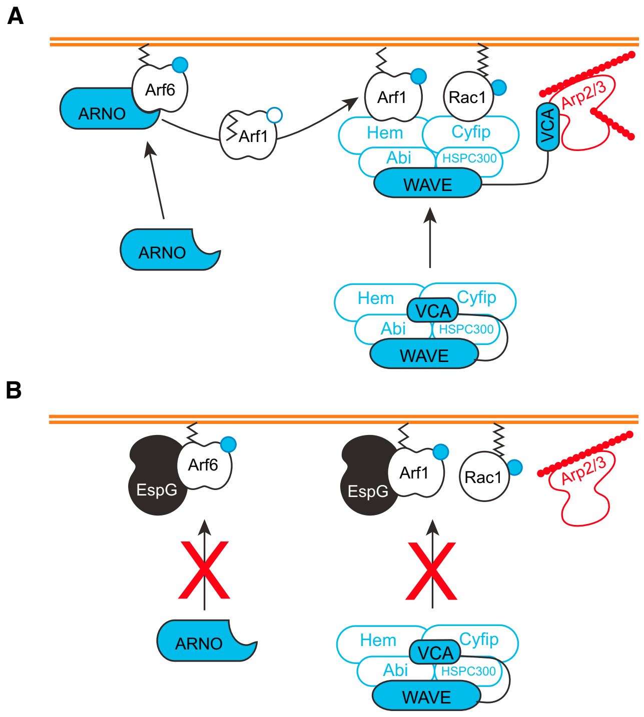

Figure 6. Model for EspG Incapacitation of the WRC

(A) Arf6 recruits and activates ARNO that activates Arf1 which consequently anchors via its exposed myristoylation moiety to the plasma membrane (brown lines). While the Arf1 binding partner remains speculative (e.g., Hem), nevertheless, membrane anchored active Arf1 and Rac1 work in synergy to recruit and activate the WRC (i.e., release of the WAVE veroprolin homology cofilin hemology acidic region [VCA] domain) that induces Arp2/3-dependent polymerization of actin filaments (red) and pathogen phagocytosis. Empty (GDP) and filled blue (GTP) circles.

(B) EspG interaction with Arf GTPases blocks actin polymerization via a dual mechanism: EspG impedes Arf6-activation of ARNO and Arf1-activation of the WRC (highlighted by the red cross).

Given the central role of Rho GTPases in phagocytosis, it is not surprising that many pathogens employ GAPs to deactivate GTPases (e.g., *Yersinia* YopE, *Pseudomonas* ExoS) (Black and Bliska, 2000; Goehring et al., 1999). Yet, EPEC and EHEC encode no known GAPs. Instead, the pathogens interfere with Rho GTPase activation through EspH that binds Rho Dbl-family GEFs to disrupt interaction with Rho GTPases (Dong et al., 2010). Here, we show that EPEC and EHEC disarm professional phagocytes through EspG that targets active Arf GTPases to block activation of ARNO and the WRC. EspG is known to sterically hinder Arf interactions with Arf GAPs and certain cellular effectors (Selyunin et al., 2011, 2014). Consistent with this view, we propose that EspG utilizes steric hindrance to prevent Arf GTPase cooperation with Rac1 in driving cytoskeleton remodeling. Thus, EPEC and EHEC likely inhibit WRC-dependent phagocytosis by nullifying both Rho activation via EspH and Arf signaling via EspG.

The study also shows that ARNO, a plasma membrane GEF of Arf1, operates in this phagocytic pathway, which is consistent with a previously reported role for Arf1 and the ARNO homolog cytohesin-1 in opsonin-dependent phagocytosis (Beemiller et al., 2006; Sendide et al., 2005). ARNO is activated at the plasma membrane by Arf6 (Stalder et al., 2011; Cohen et al., 2007), a long established regulator of phagocytosis (Niedergang et al., 2003; Zhang et al., 1998). EspG directly abrogated Arf6 recruitment and activation of ARNO thereby impeding Arf1 activation and describing a role for the Arf6-EspG interaction.

In summary, by targeting both Arf6 and Arf1 our work establishes a dual mechanism by which a single virulence effector uncouples two arms of the WRC regulatory pathway and ultimately inhibits phagocytic uptake to evade innate host defenses (depicted in our model in Figure 6).

EXPERIMENTAL PROCEDURES

Bacterial Strains

EPEC E2348/69 and EHEC EDL933 (TUV93-0 Shiga toxin deficient derivative) strains were used. Isogenic mutant EPEC ΔespG1/ ΔespG2 (Prof. Feng Shao) and EHEC ΔespG (Dr. Ken Campellone) were kind gifts. For infections, bacteria were cultured as previously described (Smith et al., 2010).

Plasmids

The following plasmids were generated by Invitrogen Gateway methodology: pET15b-espG, pGEX2T-espG, pcDNA-HA-espG (encoding effector domain residues 48–398) and pTrc99FA-espG (full length). Plasmids pET20b-Arf1, pET20b-Arf6, pET15b-Rac1, pGEX2T-ARNO-2G, and pGEX2T-GGA3-GAT$^{1–313}$ were described previously (Humphreys et al., 2013). Point mutations were introduced by site-directed mutagenesis into pET15b-espG, pGEX2T-espG (EspGΔ^{P} residues D205A, R208A; EspGΔ^{R} Q293A; EspG$\Delta^{A}\Delta^{P}$ I152S, P351A, P355A), pET20b-Arf1 or pET20b-Arf6 (mutations indicated in the text). GST- and His-tagged proteins were expressed in *E. coli* Rosetta (Novagen) at 16°C before affinity purification (Humphreys et al., 2012b).

Antibodies

Antibodies were purchased from Abcam (Rac1, ab33186; Arf1, ab58578; Arf6, ab81650; ARNO, ab56510; PAK1, ab154284), Sigma (Abi1, A5106; Actin, A2066; Cyfip, P0092; Nap1, N3788; AP1, A4200), GE Healthcare Life Sciences (GST, 27457701), QIAGEN (His, 34660) or were raised against recombinant peptides in rabbits by Diagnostics Scotland (WAVE2, amino acids 180–241). Antibodies against GST or His were used to detect EspG.

Mammalian Cell Culture and Transfection

The human monocyte-like cell line THP1s (kind gift from Prof. Gordon Dougan) and mouse macrophage-like RAW264.7 (ATCC-TIB71) cells were cultured (37°C, 5% CO_2) in RPMI-1640 or DMEM in the case of HeLa cells (ATCC-CCL-2) supplemented with 2 mM L-glutamine, 10% heat-inactivated fetal calf serum (FCS), 200 μg/mL^{-1} streptomycin, and 100 U mL^{-1} penicillin (Schwende et al., 1996). THP1s were differentiated into mature macrophage-like cells by stimulation with 100 ng/mL Phorbol 12-myristate 13-acaetate (PMA) for 3 days and then cultured for an additional day without PMA before phagocytosis assays.

Transient transfection of HeLa cells by microporation was performed using the Neon Transfection System according to the manufacturer's instructions (Invitrogen). For RNA interference, small interfering RNA (siRNA) smart pools against Arf1, Rac1, and Hem-1 or non-targeting control siRNA from Dharmacon (GE Healthcare Life Sciences) were transfected into THP1 cells with Oligofectamine transfection reagent (Invitrogen) according to manufacturer's instructions. Transfection mixture was replaced after 24 hr with complete growth medium and cells cultured 72 hr in total.

Phagocytosis Assays

Prior to infection EPEC and EHEC strains were harvested by centrifugation, washed in phosphate-buffered saline (PBS) then incubated with pH-Rodo (Thermo Fisher Scientific) before washing with Tris (pH 7.4)-buffered saline. Approximately 2 × 10^5 mammalian cells seeded onto glass coverslips were

infected with pH-Rodo-labeled bacteria (1 hr, 37°C, 5% CO_2) before washing with PBS and fixation using 4% paraformaldehyde. Fixed cells were incubated with rabbit anti-intimin (EPEC/EHEC outer membrane protein) antibodies, washed with PBS, and then incubated with anti-rabbit Alexa Flour 350 antibodies and cells visualized phalloidin-FITC-488 (Thermo Fisher Scientific) in PBS supplemented with Tx100. Phagocytosis was quantified by counting the number of extracellular bacteria labeled with intimin antibodies relative to intracellular bacteria showing pH-Rodo fluorescence using automated Volocity software (Improvision). When appropriate, cells were incubated with 25 μM Secinh3 (Merck). Immunofluorescence microscopy and images assembled as described (Humphreys et al., 2012b). All experiments were performed at least three times.

Salmonella Invasion of Non-phagocytic Host Cells

Wild-type *Salmonella enterica* serovar Typhimurium SL1344 were used to assay invasion into non-phagocytic cells as previously described (Humphreys et al., 2013). *Salmonella* encoding pM975 that expresses GFP via the SPI2 promoter when bacteria are within *Salmonella* containing vacuoles (SCVs) (Schlumberger et al., 2007) were used to infect HeLa cells (10 min), and the number of fluorescent bacteria was counted per cell microscopically.

In Vitro WRC-Dependent Actin-Based Motility

Preparation of porcine brain extract, actin-based motility by phospholipid-coated beads, and isolation of bead membrane-associated proteins have been described in detail (Hume et al., 2014). When indicated, extract or buffer containing recombinant EspG derivatives, GAT3, or ARNO was used. Quantification of comet tail length was performed on 50 comet tails per experiment using Volocity measurement software (Improvision). All experiments were performed at least three times.

Statistics

All experiments were performed at least three times. Geometric means were calculated, and significance was determined by Student's t test or one-way ANOVA followed by a post hoc Dunnett's comparison. *$p < 0.05$ was considered significant.

SUPPLEMENTAL INFORMATION

Supplemental Information includes five figures and can be found with this article online at http://dx.doi.org/10.1016/j.celrep.2016.09.039.

AUTHOR CONTRIBUTIONS

D.H., V.S., and V.K. performed experiments and wrote the paper.

ACKNOWLEDGMENTS

Thanks go to Dr. Peter Hume for technical help and advice, Rachael Stone for purifying recombinant proteins, and Dr. Eliza Wolfson for the model and graphical abstract (http://lizawolfson.co.uk/). Thanks go to Prof. Feng Shao (Bejing), Dr. Ken Campellone (Connecticut), and Prof. Gordon Dougan (Hinxton) for reagents. This work was funded by the Wellcome Trust, Medical Research Council and the Cambridge Isaac Newton Trust. V.K. and D.H. thank the Wellcome Trust for funding of a Senior Investigator Award to V.K. (101828/Z/13/Z), the MRC for a research grant to V.K. (MR/L008122/1), and a New Investigator Research Grant to D.H. (MR/M011771/1).

Received: February 26, 2016
Revised: June 24, 2016
Accepted: September 14, 2016
Published: October 11, 2016

REFERENCES

Austin, C., Boehm, M., and Tooze, S. (2002). Site-specific cross-linking reveals a differential direct interaction of class 1, 2, and 3 ADP-ribosylation factors with adaptor protein complexes 1 and 3. Biochemistry *41*, 4669–4677.

Beemiller, P., Hoppe, A.D., and Swanson, J. (2006). A phosphatidylinositol-3-kinase-dependent signal transition regulates ARF1 and ARF6 during Fcgamma receptor-mediated phagocytosis. PLoS Biol. *4*, p.e162.

Black, D.S., and Bliska, J.B. (2000). The RhoGAP activity of the Yersinia pseudotuberculosis cytotoxin YopE is required for antiphagocytic function and virulence. Mol. Microbiol. *37*, 515–527.

Caron, E., and Hall, A. (1998). Identification of two distinct mechanisms of phagocytosis controlled by different Rho GTPases. Science *282*, 1717–1721.

Celli, J., and Finlay, B.B. (2002). Bacterial avoidance of phagocytosis. Trends Microbiol. *10*, 232–237.

Celli, J., Olivier, M., and Finlay, B.B. (2001). Enteropathogenic Escherichia coli mediates antiphagocytosis through the inhibition of PI 3-kinase-dependent pathways. EMBO J. *20*, 1245–1258.

Chen, Z., Borek, D., Padrick, S.B., Gomez, T.S., Metlagel, Z., Ismail, A.M., Umetani, J., Billadeau, D.D., Otwinowski, Z., and Rosen, M.K. (2010). Structure and control of the actin regulatory WAVE complex. Nature *468*, 533–538.

Cohen, L.A., Honda, A., Varnai, P., Brown, F.D., Balla, T., and Donaldson, J.G. (2007). Active Arf6 recruits ARNO/cytohesin GEFs to the PM by binding their PH domains. Mol. Biol. Cell *18*, 2244–2253.

Cox, D., Tseng, C.C., Bjekic, G., and Greenberg, S. (1999). A requirement for phosphatidylinositol 3-kinase in pseudopod extension. J. Biol. Chem. *274*, 1240–1247.

Dean, P. (2011). Functional domains and motifs of bacterial type III effector proteins and their roles in infection. FEMS Microbiol. Rev. *35*, 1100–1125.

Dong, N., Liu, L., and Shao, F. (2010). A bacterial effector targets host DH-PH domain RhoGEFs and antagonizes macrophage phagocytosis. EMBO J. *29*, 1363–1376.

Dong, N., Zhu, Y., Lu, Q., Hu, L., Zheng, Y., and Shao, F. (2012). Structurally distinct bacterial TBC-like GAPs link Arf GTPase to Rab1 inactivation to counteract host defenses. Cell *150*, 1029–1041.

Edwards, D.C., Sanders, L.C., Bokoch, G.M., and Gill, G.N. (1999). Activation of LIM-kinase by Pak1 couples Rac/Cdc42 GTPase signalling to actin cytoskeletal dynamics. Nat. Cell Biol. *1*, 253–259.

Gautreau, A., Ho, H.Y., Li, J., Steen, H., Gygi, S.P., and Kirschner, M.W. (2004). Purification and architecture of the ubiquitous Wave complex. Proc. Natl. Acad. Sci. USA *101*, 4379–4383.

Germane, K.L., and Spiller, B.W. (2011). Structural and functional studies indicate that the EPEC effector, EspG, directly binds p21-activated kinase. Biochemistry *50*, 917–919.

Goehring, U.M., Schmidt, G., Pederson, K.J., Aktories, K., and Barbieri, J.T. (1999). The N-terminal domain of Pseudomonas aeruginosa exoenzyme S is a GTPase-activating protein for Rho GTPases. J. Biol. Chem. *274*, 36369–36372.

Goosney, D.L., Celli, J., Kenny, B., and Finlay, B.B. (1999). Enteropathogenic Escherichia coli inhibits phagocytosis. Infect. Immun. *67*, 490–495.

Hartland, E.L., and Leong, J.M. (2013). Enteropathogenic and enterohemorrhagic E. coli: Ecology, pathogenesis, and evolution. Front. Cell. Infect. Microbiol. *3*, 15.

Hume, P.J., Humphreys, D., and Koronakis, V. (2014). WAVE Regulatory Complex Activation, First Edition (Elsevier).

Humphreys, D., Davidson, A.C., Hume, P.J., Makin, L.E., and Koronakis, V. (2013). Arf6 coordinates actin assembly through the WAVE complex, a mechanism usurped by Salmonella to invade host cells. Proc. Natl. Acad. Sci. USA *110*, 16880–16885.

Humphreys, D., Liu, T., Davidson, A.C., Hume, P.J., and Koronakis, V. (2012a). Drosophila Arf1 homologue Arf79F is essential for lamellipodia formation. J. Cell Sci. *1*, 5630–5635.

Humphreys, D., Davidson, A., Hume, P.J., and Koronakis, V. (2012b). Salmonella virulence effector SopE and host GEF ARNO cooperate to recruit and activate WAVE to trigger bacterial invasion. Cell Host Microbe *11*, 129–139.

Iizumi, Y., Sagara, H., Kabe, Y., Azuma, M., Kume, K., Ogawa, M., Nagai, T., Gillespie, P.G., Sasakawa, C., and Handa, H. (2007). The enteropathogenic

E. coli effector EspB facilitates microvillus effacing and antiphagocytosis by inhibiting myosin function. Cell Host Microbe *2*, 383–392.

Kobayashi, K., Kuroda, S., Fukata, M., Nakamura, T., Nagase, T., Nomura, N., Matsuura, Y., Yoshida-Kubomura, N., Iwamatsu, A. and Kaibuchi, K. (1998). p140Sra-1 (Specifically Rac1-associated Protein) is a novel specific target for Rac1 small GTPase. *273*, 291–295.

Koronakis, V., Hume, P.J., Humphreys, D., Liu, T., Hørning, O., Jensen, O.N., and McGhie, E.J. (2011). WAVE regulatory complex activation by cooperating GTPases Arf and Rac1. Proc. Natl. Acad. Sci. USA *108*, 14449–14454.

Krause, M., and Gautreau, A. (2014). Steering cell migration: Lamellipodium dynamics and the regulation of directional persistence. Nat. Rev. Mol. Cell Biol. *15*, 577–590.

Kuai, J., Boman, A.L., Arnold, R.S., Zhu, X., and Kahn, R.A. (2000). Effects of activated ADP-ribosylation factors on Golgi morphology require neither activation of phospholipase D1 nor recruitment of coatomer. J. Biol. Chem. *275*, 4022–4032.

Lebensohn, A.M., and Kirschner, M.W. (2009). Activation of the WAVE complex by coincident signals controls actin assembly. Mol. Cell *36*, 512–524.

Macia, E., Paris, S., and Chabre, M. (2000). Binding of the PH and polybasic C-terminal domains of ARNO to phosphoinositides and to acidic lipids. Biochemistry *16*, 5893–5901.

Marchès, O., Covarelli, V., Dahan, S., Cougoule, C., Bhatta, P., Frankel, G., and Caron, E. (2008). EspJ of enteropathogenic and enterohaemorrhagic Escherichia coli inhibits opsono-phagocytosis. Cell. Microbiol. *10*, 1104–1115.

May, R.C., Caron, E., Hall, A., and Machesky, L.M. (2000). Involvement of the Arp2/3 complex in phagocytosis mediated by FcγR or CR3. Nat. Cell Biol. *2*, 246–248.

Miki, H., Suetsugu, S., and Takenawa, T. (1998). WAVE, a novel WASP-family protein involved in actin reorganization induced by Rac. EMBO J. *17*, 6932–6941.

Nie, Z., Hirsch, D.S., and Randazzo, P.A. (2003). Arf and its many interactors. Curr. Opin. Cell Biol. *15*, 396–404.

Niedergang, F., Colucci-Guyon, E., Dubois, T., Raposo, G., and Chavrier, P. (2003). ADP ribosylation factor 6 is activated and controls membrane delivery during phagocytosis in macrophages. J. Cell Biol. *161*, 1143–1150.

Palamidessi, A., Frittoli, E., Garré, M., Faretta, M., Mione, M., Testa, I., Diaspro, A., Lanzetti, L., Scita, G., and Di Fiore, P.P. (2008). Endocytic trafficking of Rac is required for the spatial restriction of signaling in cell migration. Cell *134*, 135–147.

Park, H., Staehling-Hampton, K., Appleby, M.W., Brunkow, M.E., Habib, T., Zhang, Y., Ramsdell, F., Liggitt, H.D., Freie, B., Tsang, M., et al. (2008). A point mutation in the murine Hem1 gene reveals an essential role for Hematopoietic protein 1 in lymphopoiesis and innate immunity. J. Exp. Med. *205*, 2899–2913.

Pils, S., Kopp, K., Peterson, L., Delgado Tascón, J., Nyffenegger-Jann, N.J., and Hauck, C.R. (2012). The adaptor molecule Nck localizes the WAVE complex to promote actin polymerization during CEACAM3-mediated phagocytosis of bacteria. PLoS ONE *7*, e32808.

Quitard, S., Dean, P., Maresca, M., and Kenny, B. (2006). The enteropathogenic Escherichia coli EspF effector molecule inhibits PI-3 kinase-mediated uptake independently of mitochondrial targeting. Cell. Microbiol. *8*, 972–981.

Ren, X., Farías, G.G., Canagarajah, B.J., Bonifacino, J.S., and Hurley, J.H. (2013). Structural basis for recruitment and activation of the AP-1 clathrin adaptor complex by Arf1. Cell *152*, 755–767.

Santos, A.S., and Finlay, B.B. (2015). Bringing down the host: Enteropathogenic and enterohaemorrhagic Escherichia coli effector-mediated subversion of host innate immune pathways. Cell. Microbiol. *17*, 318–332.

Sarantis, H., and Grinstein, S. (2012). Subversion of phagocytosis for pathogen survival. Cell Host Microbe *12*, 419–431.

Schlumberger, M.C., Käppeli, R., Wetter, M., Müller, A.J., Misselwitz, B., Dilling, S., Kremer, M., and Hardt, W.D. (2007). Two newly identified SipA domains (F1, F2) steer effector protein localization and contribute to Salmonella host cell manipulation. Mol. Microbiol. *65*, 741–760.

Schwende, H., Fitzke, E., Ambs, P., and Dieter, P. (1996). Differences in the state of differentiation of THP-1 cells induced by phorbol ester and 1,25-dihydroxyvitamin D3. J. Leukoc. Biol. *59*, 555–561.

Seastone, D.J., Harris, E., Temesvari, L.A., Bear, J.E., Saxe, C.L., and Cardelli, J. (2001). The WASp-like protein scar regulates macropinocytosis, phagocytosis and endosomal membrane flow in Dictyostelium. J. Cell Sci. *114*, 2673–2683.

Selyunin, A.S., Sutton, S.E., Weigele, B.A., Reddick, L.E., Orchard, R.C., Bresson, S.M., Tomchick, D.R., and Alto, N.M. (2011). The assembly of a GTPase-kinase signalling complex by a bacterial catalytic scaffold. Nature *469*, 107–111.

Selyunin, A.S., Reddick, L.E., Weigele, B.A., and Alto, N.M. (2014). Selective protection of an ARF1-GTP signaling axis by a bacterial scaffold induces bidirectional trafficking arrest. Cell Rep. *13*, 878–891.

Sendide, K., Reiner, N.E., Lee, J.S., Bourgoin, S., Talal, A., and Hmama, Z. (2005). Cross-talk between CD14 and complement receptor 3 promotes phagocytosis of mycobacteria: Regulation by phosphatidylinositol 3-kinase and cytohesin-1. J. Immunol. *174*, 4210–4219. http://www.jimmunol.org/content/174/7/4210.full.pdf.

Smith, K., Humphreys, D., Hume, P.J., and Koronakis, V. (2010). Enteropathogenic Escherichia coli recruits the cellular inositol phosphatase SHIP2 to regulate actin-pedestal formation. Cell Host Microbe *7*, 13–24.

Stalder, D., Barelli, H., Gautier, R., Macia, E., Jackson, C.L., and Antonny, B. (2011). Kinetic studies of the Arf activator Arno on model membranes in the presence of Arf effectors suggest control by a positive feedback loop. J. Biol. Chem. *286*, 3873–3883.

Young, J.C., Clements, A., Lang, A.E., Garnett, J.A., Munera, D., Arbeloa, A., Pearson, J., Hartland, E.L., Matthews, S.J., Mousnier, A., et al. (2014). The Escherichia coli effector EspJ blocks Src kinase activity via amidation and ADP ribosylation. Nat. Commun. *5*, 5887.

Zhang, Q., Cox, D., Tseng, C.C., Donaldson, J.G., and Greenberg, S. (1998). A requirement for ARF6 in Fcgamma receptor-mediated phagocytosis in macrophages. J. Biol. Chem. *273*, 19977–19981.

Cell Reports, Volume *17*

Supplemental Information

Inhibition of WAVE Regulatory Complex Activation by a Bacterial Virulence Effector Counteracts Pathogen Phagocytosis

Daniel Humphreys, Vikash Singh, and Vassilis Koronakis

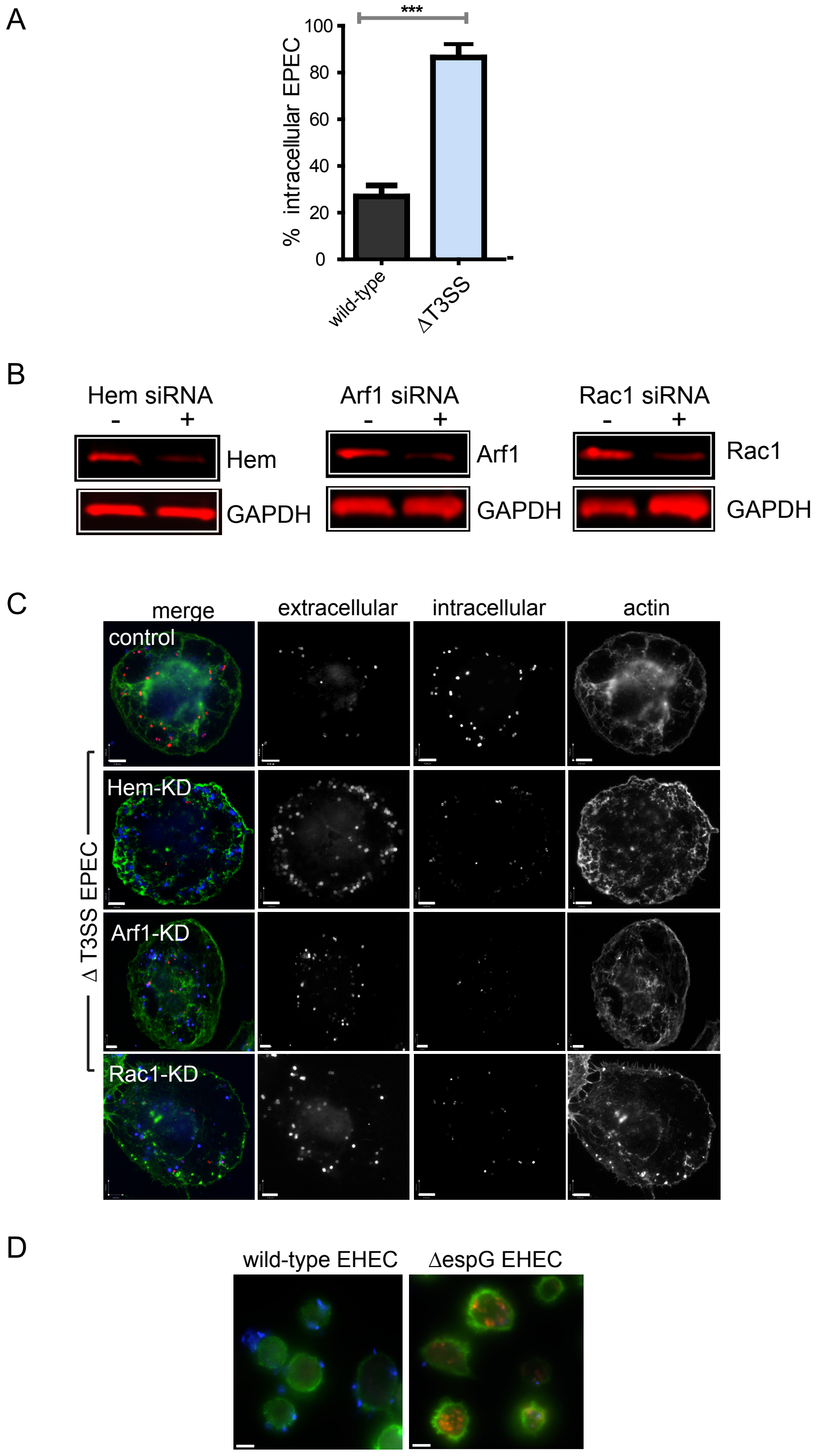

Figure S1. Anti-phagocytosis of EPEC and EHEC (related to Fig 1).

(**A**) Phagocytosis of wild-type and ΔT3SS EPEC in RAW264.7 mouse macrophage cells. Error bars represent ± SEM. Asterisks indicate a significant difference from wild-type. (**B**) Knockdown of Hem, Arf1 and Rac1 in THP1s following siRNA transfection from Figure 1. Immunoblotting of whole cell lysates with antibodies recognising Hem, Arf1 and Rac1 and GAPDH as a loading control. (**C**) Images showing phagocytosis of EPEC$^{\Delta T3SS}$ (ΔT3SS) in knockdown cells from (B). Intracellular bacteria (red), extracellular bacteria (blue) and host cells (actin). Scale bars 6μm. Quantification shown in Fig 1C. (**D**) Phagocytosis of wild-type or ΔespG EHEC by THP1s. Intracellular bacteria (red), extracellular bacteria (blue) and host cells (actin). Scale bars 6μm. All experiments in Fig S1 were performed in triplicate at least three times.

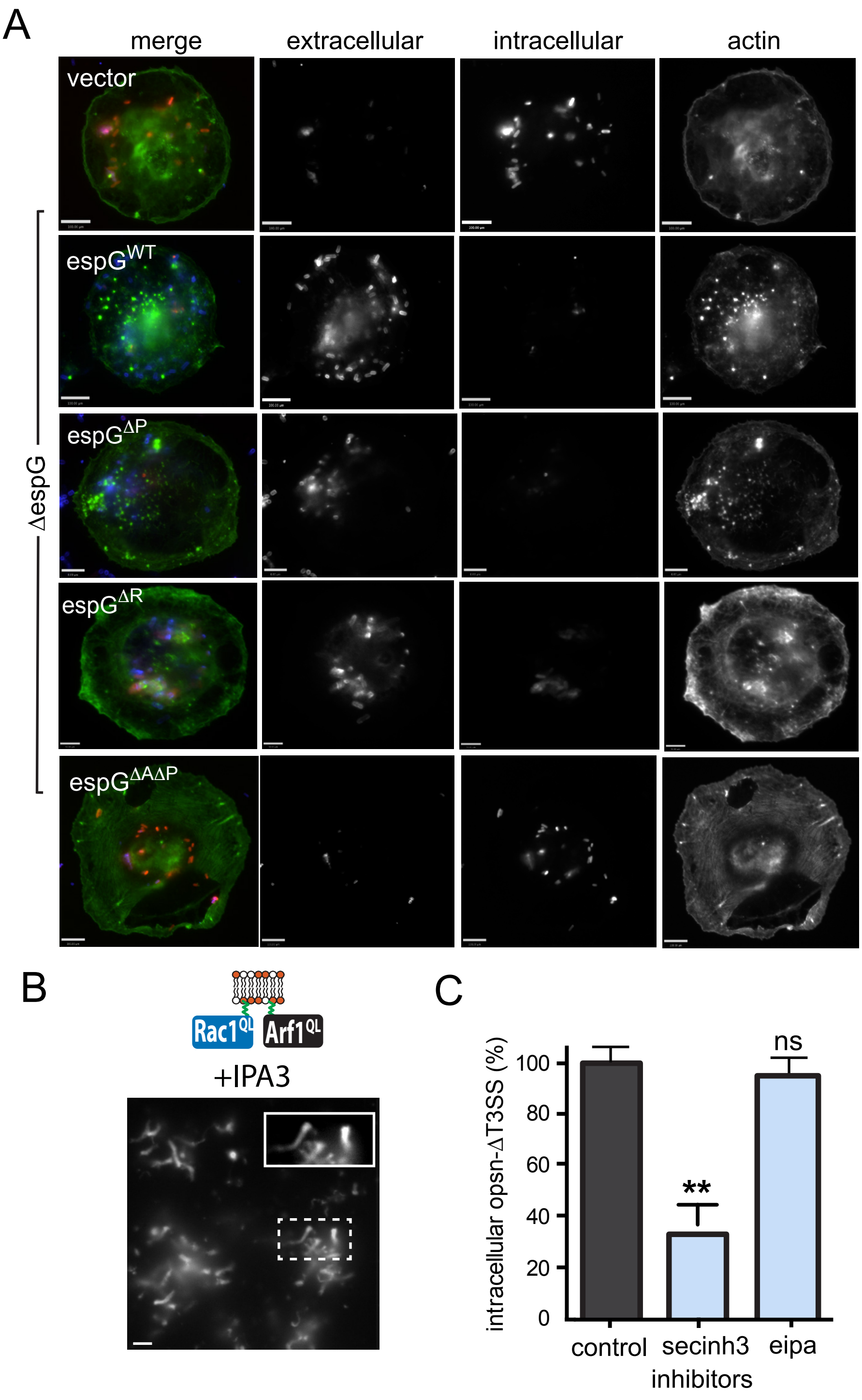

Figure S2. EspG counteracts phagocytosis by binding Arf GTPases (related to Fig 2 and Fig 3).

(**A**) Images showing phagocytosis of $EPEC^{\Delta espG}$ (ΔespG) expressing a control vector or the vector encoding espG variants, namely wild-type (WT), or mutants in binding Rab ($EspG^{\Delta R}$), PAK ($EspG^{\Delta P}$) or both Arf and PAK ($EspG^{\Delta A\Delta P}$). Scale bars 6μm. Quantification shown in Fig 2F. (**B**) WRC-dependent actin-based motility directed by $Arf1^{QL}$ and $Rac1^{QL}$ in extract containing the inhibitor of PAK activation IPA3. (**C**) THP1 phagocytosis of $EPEC^{\Delta T3SS}$ (ΔT3SS) in the presence of opsonising human serum and inhibitors of ARNO (secinh3) or macropinocytosis (eipa). Error bars represent ± SEM. Asterisks indicate a significant difference from control (black bars). Not significant (ns). All experiments in Fig S2 were performed at least three times.

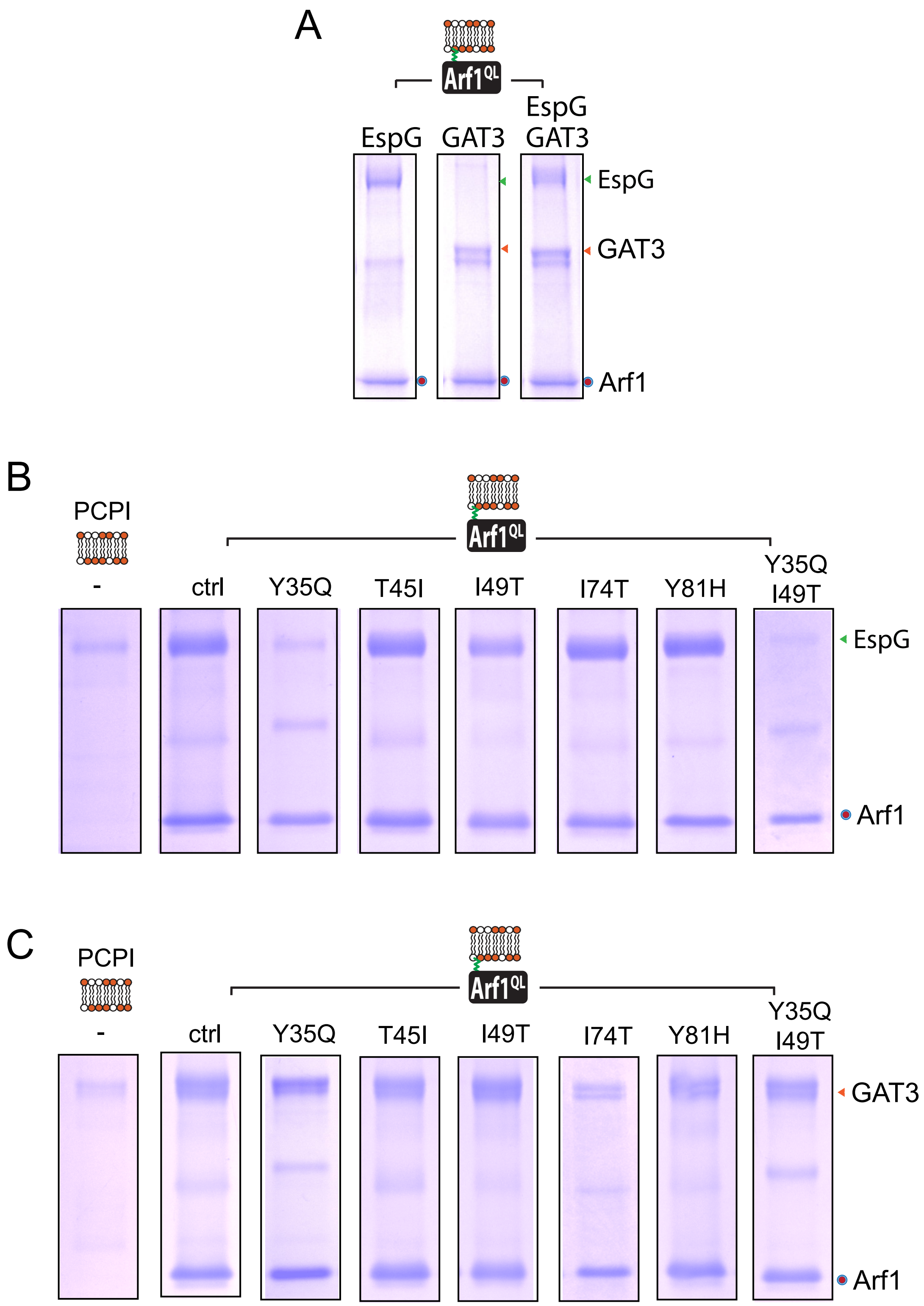

Figure S3. Arf1 interaction with EspG and GAT3 (related to Fig 4).

(**A**) Membrane-anchored Arf1^{QL} interaction with GST-EspG (green arrow) or His-GAT3 (red arrow), or both in combination, in buffer. (**B**) Interaction of GST-EspG with PCPI-membranes alone (-) as a control or with membranes anchored with Arf1^{QL} (ctrl) or Arf1^{QL} derivatives incorporating mutations within the alpha-1 helix (Y35Q), switch 1 (T45I, I49T) or switch 2 (I74T, Y81H) domain as indicated. The results show that EspG binds Arf1 residue Y35 and I49, albeit to a lesser extent (**C**) Experiment performed as (B) with GST-GAT3. The results show that GAT3 weakly interacts with I74. All experiments in Fig S3 were performed at least three times.

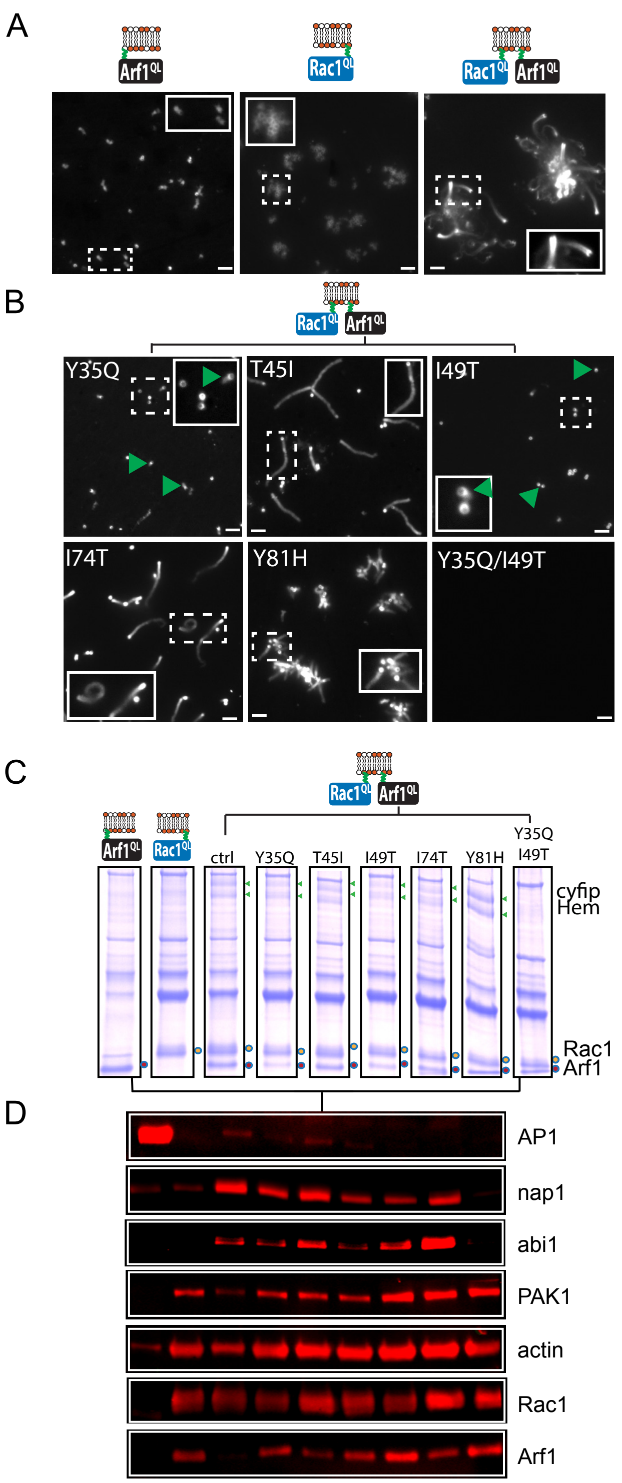

Figure S4. Molecular basis of Arf1 cooperation with Rac1 in WRC recruitment and activation (related to Fig 5).

(**A**) WRC-dependent actin-based motility directed by Arf1^{QL} or Rac1^{QL} alone, or in combination. Insets magnify actin-comet tails. Scale bars 5μm. The results show that only Arf1^{QL} or Rac1^{QL} co-anchored at the membrane activate the WRC (**B**) WRC-dependent actin-based motility directed by Rac1^{QL} in combination with Arf1^{QL} derivatives incorporating mutations within the alpha-1 helix (Y35Q), switch 1 (T45I, I49T) or switch 2 (I74T, Y81H) domain as indicated. Scale bars 5μm. The data show that Arf1 residues Y35 and I49 are required for WRC activation (**C**) Proteins recruited by membrane-anchored Arf1^{QL} or Rac1^{QL} alone, or Rac1^{QL} in combination with Arf1^{QL} containing indicated mutations. Green arrows indicate cyfip and Hem. (**D**) Immunoblotting of samples from (C) with indicated antibodies (right). The data in (C) and (D) show that Arf1 residues Y35 and I49 are required for WRC recruitment (i.e. Y35Q/I49T). All experiments in Fig S4 were performed at least three times.

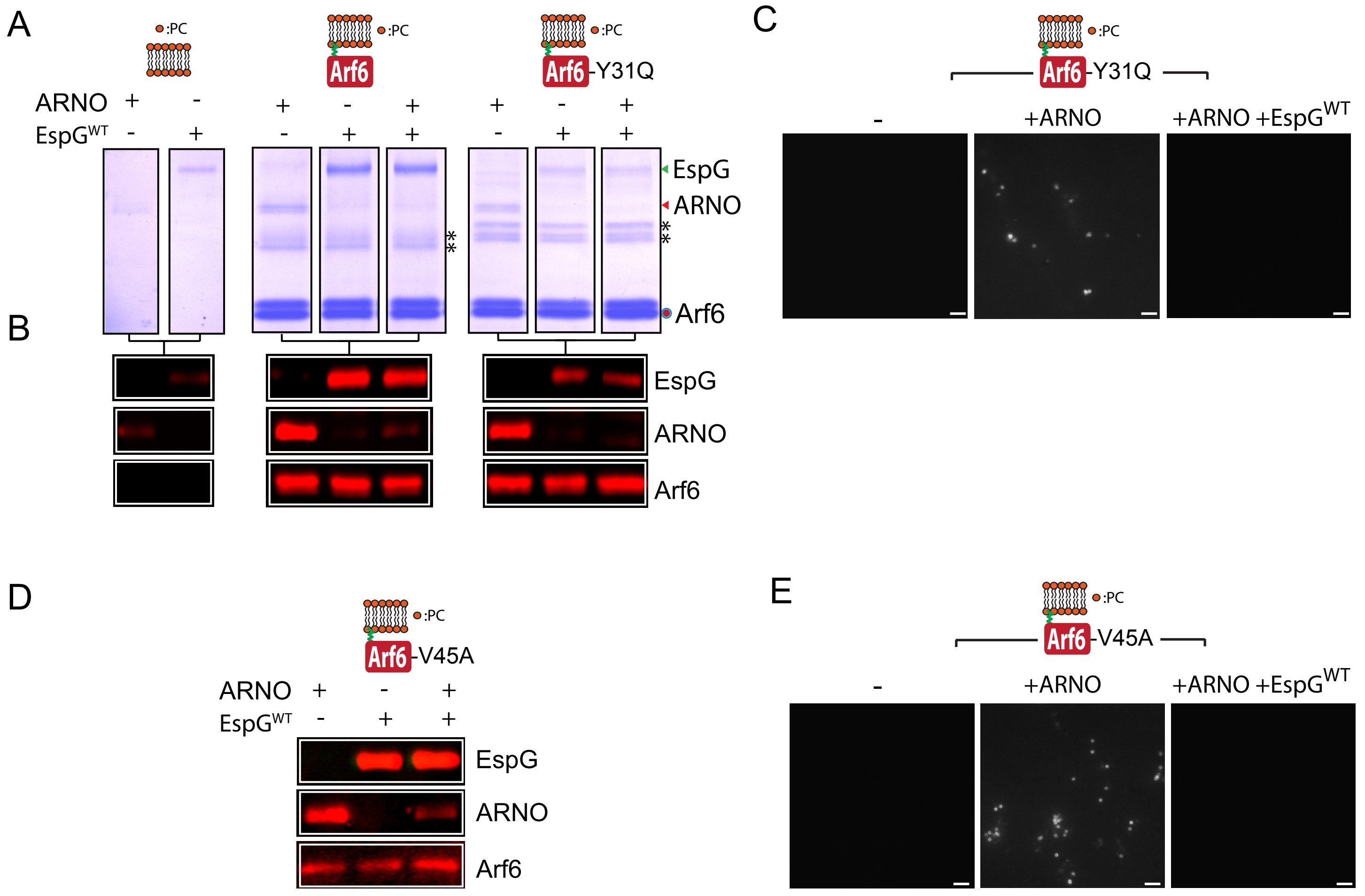

Figure S5. Ability of Arf6 mutants to interact with EspG and ARNO, and activate the WRC (related to the discussion).

(**A**) The interaction of PC membranes alone (left) or membranes anchored with GTPγS-loaded wild-type or Y31Q Arf6 with His-ARNO (red arrow) or GST-EspG (green arrow) alone, or both in combination, in buffer. Asterisks indicate Arf6 dimers. To assess the influence of the Arf6-Y31Q mutation, PC and Arf6 controls shown left are duplicated from Fig 3G for clarity. (**B**) Immunoblotting of samples from (A) with indicated antibodies against GST (EspG), ARNO and Arf6 (right). To assess the influence of the Arf6-Y31Q mutation, PC and Arf6 controls shown left are duplicated from Fig 3G for clarity. (**C**) WRC-dependent actin assembly via membrane-anchored $Arf6^{GTP\gamma S}$-Y31Q (depicted in cartoon) in cell-free extract alone or in extract containing recombinant ARNO in the presence or absence of recombinant $EspG^{WT}$. Collectively, the results from (A, B and C) show that the Y31Q mutation attenuates interactions with EspG but interactions at the membrane are still sufficient for EspG to impede ARNO-Ar6-Y31Q associations in buffer (A, B) and WRC activation in extract (C). (**D**) Immunoblotting of proteins
